# Arvcf Dependent Adherens Junction Stability is Required to Prevent Age-Related Cortical Cataracts

**DOI:** 10.3389/fcell.2022.840129

**Published:** 2022-07-06

**Authors:** Jessica B. Martin, Kenneth Herman, Nathalie S. Houssin, Wade Rich, Matthew A. Reilly, Timothy F. Plageman

**Affiliations:** ^1^ College of Optometry, The Ohio State University, Columbus, OH, United States; ^2^ Department of Biomedical Engineering, College of Engineering, The Ohio State University, Columbus, OH, United States; ^3^ Department of Ophthalmology and Visual Science, College of Medicine, The Ohio State University, Columbus, OH, United States

**Keywords:** ARVCF, lens, fiber cell, adherens junction, N-cadherin, catenin, nanocluster, age-dependent cortical cataracts

## Abstract

The etiology of age-related cortical cataracts is not well understood but is speculated to be related to alterations in cell adhesion and/or the changing mechanical stresses occurring in the lens with time. The role of cell adhesion in maintaining lens transparency with age is difficult to assess because of the developmental and physiological roles that well-characterized adhesion proteins have in the lens. This report demonstrates that Arvcf, a member of the p120-catenin subfamily of catenins that bind to the juxtamembrane domain of cadherins, is an essential fiber cell protein that preserves lens transparency with age in mice. No major developmental defects are observed in the absence of *Arvcf*, however, cortical cataracts emerge in all animals examined older than 6-months of age. While opacities are not obvious in young animals, histological anomalies are observed in lenses at 4-weeks that include fiber cell separations, regions of hexagonal lattice disorganization, and absence of immunolabeled membranes. Compression analysis of whole lenses also revealed that Arvcf is required for their normal biomechanical properties. Immunofluorescent labeling of control and Arvcf-deficient lens fiber cells revealed a reduction in membrane localization of N-cadherin, β-catenin, and αN-catenin. Furthermore, super-resolution imaging demonstrated that the reduction in protein membrane localization is correlated with smaller cadherin nanoclusters. Additional characterization of lens fiber cell morphology with electron microscopy and high resolution fluorescent imaging also showed that the cellular protrusions of fiber cells are abnormally elongated with a reduction and disorganization of cadherin complex protein localization. Together, these data demonstrate that Arvcf is required to maintain transparency with age by mediating the stability of the N-cadherin protein complex in adherens junctions.

## Introduction

The ability of the lens of the eye to focus a clear image on the retina depends on its transparency and refractive properties. Age-related pathologies that affect this function are extremely common and result from opacities that can occur in the center or nucleus of the lens (nuclear cataracts) and within its outer margins or cortical region (cortical cataracts) (Beebe et al., 2010; Khairallah et al., 2015). While age-related nuclear cataracts are caused by an accumulation of misfolded, chemically modified and aggregated proteins that eventually overwhelm the lens’ physiological protective mechanisms to preserve transparency (Truscott and Friedrich, 2019), the etiology of cortical cataracts is less understood. The bulk of the lens mass is made up of extremely long arched lens fiber cells that extend from the posterior end of the lens to its anterior margin where they meet a cap of relatively flat lens epithelial cells (Bassnett et al., 2011; Cvekl and Ashery-Padan, 2014). Cortical cataracts are typified by an initial disruption to the structure of localized groups of fiber cells near the lens equator which lie behind the iris and are difficult to clinically detect. These disruptions are characterized by opacities coincident with cellular breaks, folds, and localized swelling, accumulations of intracellular membranous vesicles, calcium ion deposition, and are generally thought to be more damaging to lens fiber cells than what occurs during age-related nuclear cataracts (Vrensen and Willekens, 1990; Brown et al., 1993; Michael et al., 2008). Vision is negatively affected when the opacities radiate along the length of lens fiber cells anteriorly and/or posteriorly and enter the light path. Eventually, the region of opacities and cellular disruption can extend beyond the cortical regions and into the lens nucleus further eroding vision (Beebe et al., 2010). Although the cellular disruptions occurring during cortical cataracts are well documented, the initiating factors underlying these events are not well understood.

Given that opacities begin in the cortical region of the lens, this location is a logical place to investigate the etiology of cortical cataracts. One of the morphological attributes of lens fiber cells within this region is the presence of a number of cellular protrusions that interlock with their neighbors. These protrusions can be found along the entire length of the fiber cells both within the broad bicellular membrane regions (referred to as ball-and-socket protrusions) and at the confluence of three cells (called interlocking protrusions). Furthermore, the interlocking protrusions emanate from larger, higher-order undulations of the cell membrane with puzzle-piece-like structures called paddles (Blankenship et al., 2007). All of these structures are largely absent from in the outer-most fiber cells but have increased size and abundance along cells found deeper within the lens cortex (Bassnett et al., 2011). The interlocking nature of these structures suggests they may provide mechanical rigidity that may resist lens deformation. Supporting this notion is the presence of a high density of protein complexes with adhesive function within these protrusions. Connexin proteins, the subunits of gap junctions, have adhesive ability and are concentrated within in ball and socket protrusions (Biswas et al., 2010; Wang et al., 2016; Hu et al., 2017). Similarly, Aquaporin-0 provides adhesiveness between cells and are concentrated in interlocking protrusions (Gonen et al., 2004; Lo et al., 2014; Varadaraj and Kumari, 2018). Mutations in these genes are well known to be associated with cases of congenital nuclear cataracts in humans and animal models and have significant disruptions of the lens fiber cell protrusion structures (Shiels and Bassnett, 1996; Berthoud et al., 2014; Lo et al., 2014; Wang et al., 2016; Sun et al., 2021). Many of the adhesive proteins that make up the adherens junctional complex such as N-cadherin, β-catenin, α-N-catenin, and Nectin-3 are also abundant in fiber cells but localization of cadherin complex proteins to protrusion structures in adult fiber cells has not been demonstrated (Maisel and Atreya, 1990; Bagchi et al., 2002; Lachke et al., 2012). These proteins are all required for normal lens development or viability. As such, they also commonly have significantly disrupted lens fiber cells from an early age which hinders progress on determining their role in protrusions or in age-related cataracts (Abe et al., 2004; Smith et al., 2005; Cain et al., 2008; Pontoriero et al., 2009; Lachke et al., 2012; Logan C. M. et al., 2017).

Although many cadherin-complex proteins have been analyzed, a role for the catenins among the p120-catenin subfamily have not yet been investigated in a mature lens. This class of catenins stabilizes cadherin complexes by binding to a cytosolic domain distinct from β-catenin/plakoglobin and plakophilin and modulates cytoskeletal architecture through the regulation of GTPase activity (Anastasiadis and Reynolds, 2001; McCrea and Park, 2007; Menke and Giehl, 2012). The core members of this subfamily include p120-catenin, Arvcf (Armadillo gene deleted in velocardiofacial syndrome), δ-catenin, and p0071, all of which, except for p0071, have been detected in mature lens tissue (Straub et al., 2003; Jun et al., 2012; Maddala and Rao, 2017). Conditional ablation of p120-catenin causes abnormalities in the shape of the embryonic lens placode, but a later role was not determined (Lang et al., 2014). Mutations in the δ-catenin encoding gene are associated with cortical cataracts and high myopia in the human population, however loss of function studies for this gene in animal models have not yet been performed (Li et al., 2011; Jun et al., 2012). The human *ARVCF* gene derives its name from its location within the critical region missing in Velo-cardio-facial-syndrome (VCFS), a chromosomal disorder missing a portion of chromosome 22 (Shprintzen, 2008). A large majority of patients with VCFS (∼70%) have one of several types of ocular abnormalities that include retinal tortuosity, small optic discs, refractive errors, microphthalmia, and occasionally cortical cataracts. (Beemer et al., 1986; Mansour et al., 1987; Abel and O'Leary, 1997; Casteels et al., 2008; Gokturk et al., 2016; Allegrini et al., 2017). However, an analysis of the eyes of Arvcf deficient mice has not occurred.

In this study, the function of Arvcf was analyzed in the mouse lens for the first time. It was observed that Arvcf is the most widely expressed member of the p120-catenin subfamily within the lens and Arvcf-deficient lenses develop age-related cortical cataracts. To investigate what may lead to a loss of transparency, conventional fluorescent- and high-resolution confocal-microscopy were performed on lens tissue. A significant reduction in N-cadherin, β-catenin, and α-N-catenin localization to bicellular, lens fiber membranes occurs in the absence of Arvcf. Collectively, the data described herein demonstrate that the stabilization of the cadherin complex within adherens junctions is essential for maintaining the transparency of the lens with age.

## Results

### Arvcf is an Abundant Catenin Expressed in Lens Fiber Cells

**FIGURE 1:**
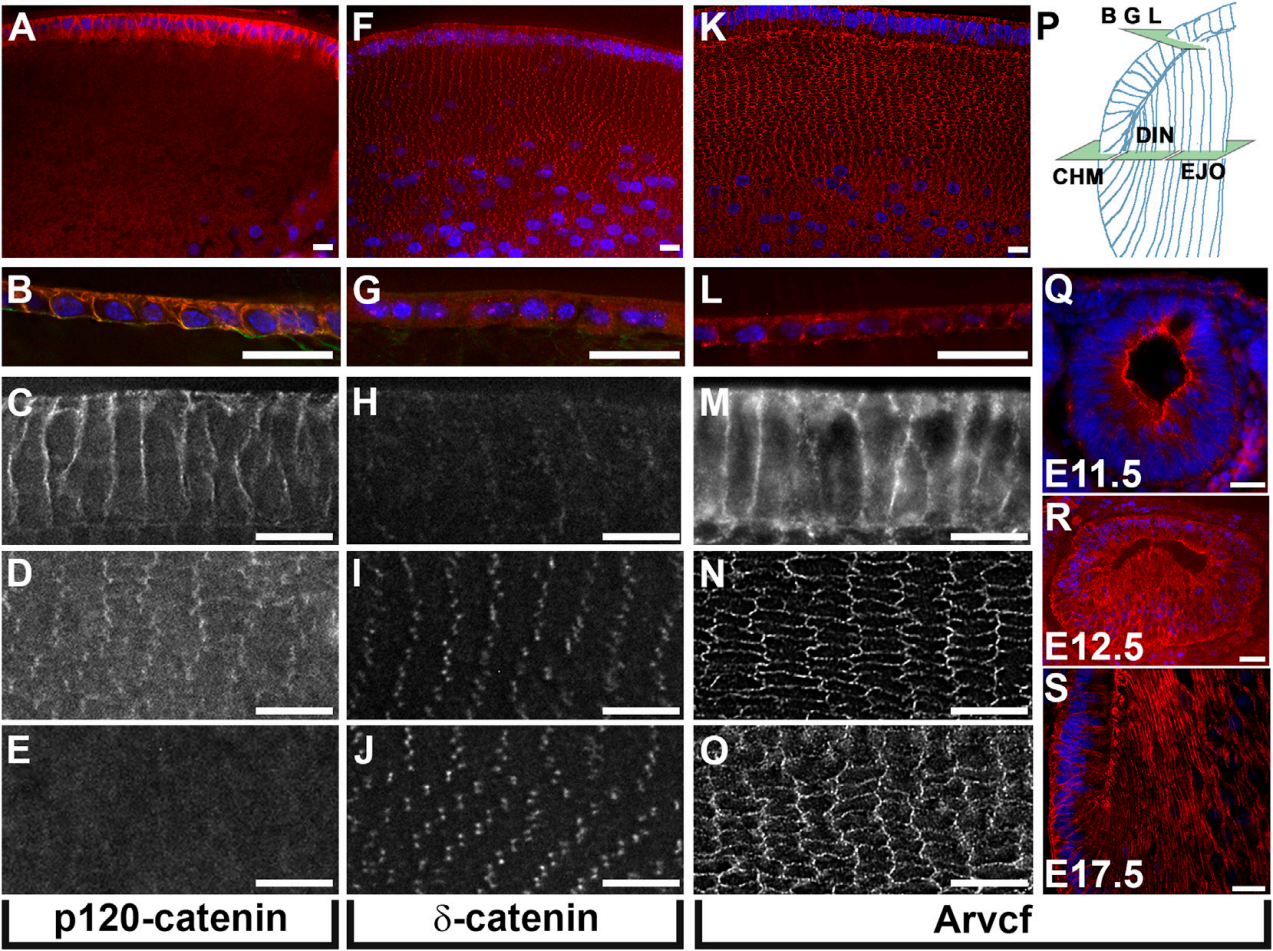
Lens expression of p120-catenin subfamily proteins. **(A–O)** Immunofluorescent labeling of equatorially cryosectioned juvenile mouse lenses (P7) using antibodies specific for p120-catenin **(A–E)** δ-catenin **(F–J)**, and Arvcf **(K–O)**. **(P)** Diagram of a portion of the lens indicating the plane of section and approximate location within the lens for **(A–O)**. Expression within the lens epithelium **(B,G,L)**, the outermost elongating lens fiber cells near the fulcrum **(C,H,M)**, the outermost cortical lens fiber cells within 50 microns of the surface **(D,I,N)**, and lens fiber cells 100 microns from the surface **(E,J,O)** is depicted. Note that p120-catenin and Arvcf but not δ-catenin are localized to the junctions of lens epithelial cells and that δ-catenin and Arvcf are much stronger than p120-catenin in lens fiber cells. Arvcf is also localized strongly to bicellular lens fiber cell junctions while δ-catenin appears restricted to lens fiber cell tricellular junctions. **(Q–S)** Immunofluorescent labeling of transversely cryosectioned mouse embryos at the indicated ages immunofluorescently labeled for Arvcf (red). Blue signal for all panels represents Hoechst labeling of nuclei. A Plan-Apochromat ×40/1.3 Oil objective with wide-field fluorescent microscope was utilized. Scalebars = 25 microns.

Because the catenins of p120-catenin subfamily play a key role in cell adhesion but have not been investigated in the adult lens, the localization of p120-catenin, Arvcf and δ-catenin were examined. Immunolabeling histological cryosections of juvenile mouse lenses (P7) revealed that p120-catenin is strongly localized to the cell membranes of the youngest elongating lens fiber cells and the lens epithelium (Figures 1A–C). However, its expression appears greatly reduced in lens fiber cells located immediately adjacent to the outermost lens fiber cells (Figure 1D) and is almost undetectable among deeper fiber cells (Figure 1E). In contrast, δ-catenin is barely detectable in the lens epithelium and superficial lens fiber cells (Figures 1F–H). δ-catenin is strongly localized to the tricellular junctions of cortical lens fiber cells (Figures 1I,J). Relatively strong Arvcf signal is detected in the bicellular junctions of both lens epithelial cells and lens fiber cells (Figures 1K–O). Developmental expression of Arvcf is also detected as early as E11.5 throughout the lens vesicle cell membranes (Figure 1Q) and continues throughout development. Strong localization along the length of primary fiber cell lateral membranes at E12.5 (Figure 1R) and secondary fiber cells at E17.5 is also observed (Figure 1S).

### Cortical Cataracts and Alterations to Lens Properties Occur in Aged Arvcf Mutant Mice

Given the strong expression of Arvcf in lens fiber cells it was hypothesized that it may be important for lens development and/or transparency. To test this, a mouse line harboring a targeted allele of *Arvcf* (*Arvcf* ^*tm1e(EUCOMM)Wtsi*^) was acquired from a commercial repository and analyzed. The allele was designed such that it disrupts endogenous RNA splicing by the insertion of a lacZ/neomycin cassette that is flanked by a splice acceptor and termination sequence 5’ of the first coding exon common to all known transcripts (Supplementary Figure S1A). Homozygous mice with two copies of the allele were generated and are usually morphologically indistinguishable from heterozygous or wild-type littermates but are born at a reduced Mendelian frequency (60/340 or 17.6% vs. the Mendelian 25%). The prevalence of heterozygous (189/340 or 55.6 vs. 50%) and wild-type (91/340 or 26.8 vs. 25%) mice are both slightly increased from expectations. The exception to a normal appearance was that some homozygous mice (10/60 or 16.7%) were noticeably runted at frequencies greater than heterozygous (6/189 or 3.2%) and wild-type mice (2/91 or 2.2%). To determine if homozygous animals were dying embryonically due to major developmental disruptions, embryos were collected from timed matings at E15.5. However, all embryos observed appeared morphologically normal, were present in Mendelian ratios, and their eyes and lenses appeared to have relatively normal morphology (Supplementary Figures S1C–F). It is yet unclear why these animals are underrepresented at birth but it could be a result of matriphagy before pups are observed.

**FIGURE 2:**
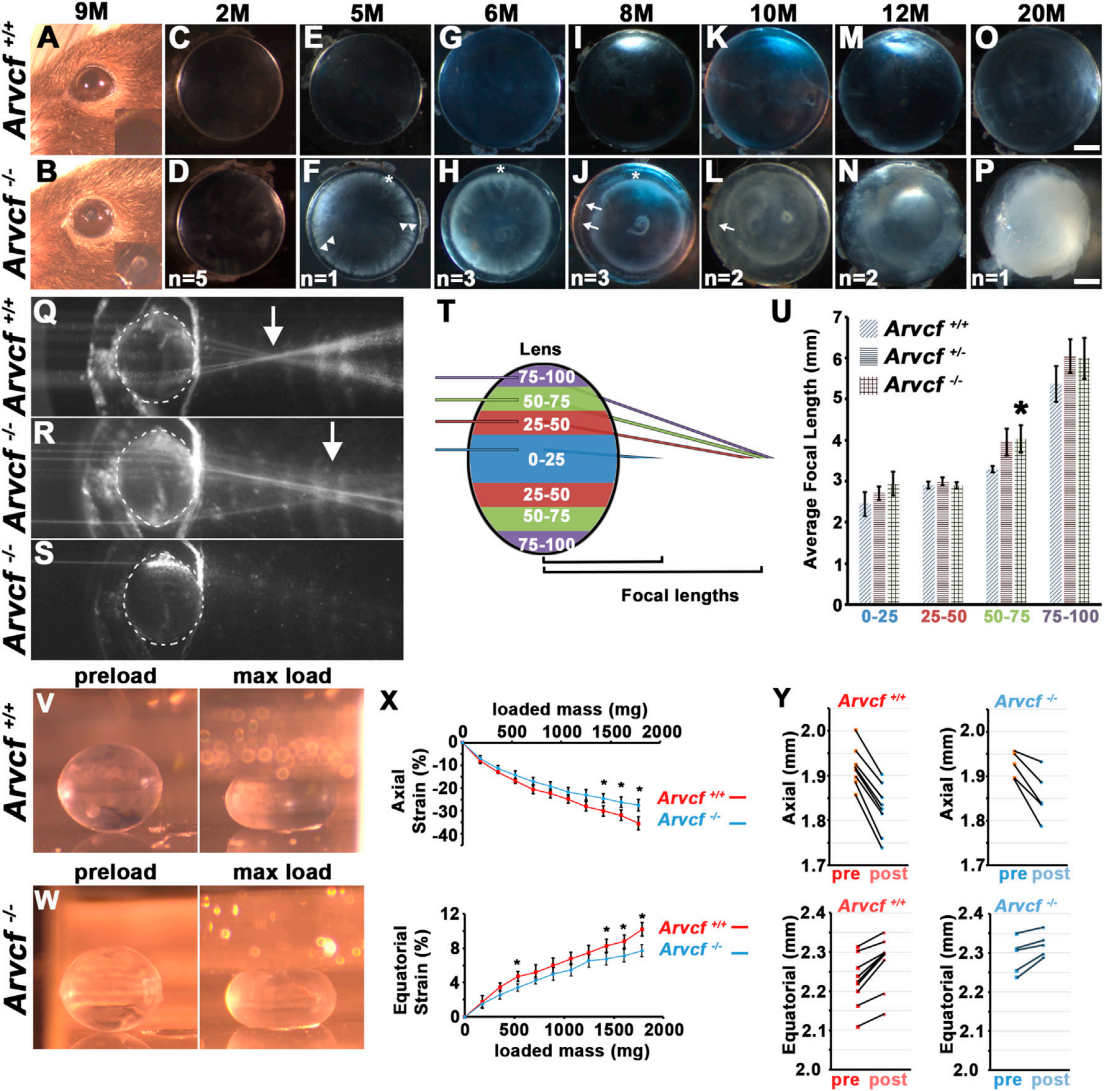
Arvcf is required to maintain lens transparency, refraction, and biomechanical properties. **(A–B)** Images of the eye of 9 month-old mice with the indicated genotypes. The inset is a magnified region of the pupil. Note the presence of an anterior polar cataract is visible in **(B)**. **(C–P)** Representative images of lenses placed on a dark background that were extracted from the eyes of mice with the indicated genotype. The asterisks mark regions of the superficial lens that remain transparent, the arrowheads indicate regions of cortical spoking, and the arrows indicate additional regions of cortical opacity within the initially transparent and expanding region. The number on the bottom of each image are the number of animals examined at each age. Note that when opacities were observed they were always bilateral. **(Q–S)** Compilation of several overlaid images taken of a single representative lens with the paths of several beams of light from a He/Ne laser aimed at several positions along the equatorial axis. Note that the approximate location of several focal lengths are visible and that the arrow denotes that Arvcf mutant lenses have a visibly longer focal length. When aimed at a region of opacity, the beam of light is blocked **(S)**. **(T)** Diagram of the laser beam light path positional groups used for quantitation of the focal length measurements. Measurements from each colored region were grouped together. **(U)** The graph depicts the mean focal lengths and standard error for laser beam paths that were calculated from all of the measurements taken from multiple positions (indicated by the colored regions in **(T)**). The asterisk indicates a statistically significant difference from the *Arvcf* ^*+/+*^ measurements *p* < 0.05 (*Arvcf* ^*+/+*^: n = 11, *Arvcf* ^*+/-*^: n = 18, *Arvcf* ^*−/−*^n = 6). Scalebar = 500microns. **(V)**) Representative stereomicroscope images of 60-day old mouse *Arvcf* ^*+/+*^ (top) and *Arvcf* ^*−/−*^ (bottom) lenses taken through a 45° mirror unloaded (left) or loaded with 10 coverslips (right). **(X)** The graph depicts the mean strain in the axial **(E)** or equatorial dimension following the addition of 1–10 coverslips placed on the anterior pole. The error bars represent the standard error and the asterisks denote data points that are significantly different than the control (*p* < 0.05). Note that the *Arvcf* ^*−/−*^ lenses do not deform as readily as the control in both dimensions. **(Y)** Graphs of the axial (top) and equatorial (bottom) dimensions of control (left) and mutant lenses (right) before coverslips were added (pre) and 2 min following their removal (post). Note that after this time period the lenses have not returned to their pre-load dimensions. Each black line depicts the change between two time points of the same lens from a single experiment.

Cataracts were not observed in young adult mice, however, aged mice developed noticeable opacities (Figures 2A,B). To examine the progression of these opacities, lenses were extracted from mice at increasing ages and imaged with a dark background (Figures 2C–P). Lenses from control and homozygous mice were completely transparent at 2 months of age (Figures 2C,D) but by 5 months, opacities were observed that appeared similar to clinical observations of cortical spoking in cortical cataracts (Figures 2E,F; Supplementary Figures S2A,B, arrowheads). These opacities were not present in heterozygous or wild-type littermate control animals (Figures 2E,G,I,K,M,O), the outer most lens fiber cells (Figures 2F,H,J, asterisks), or initially within the lens nucleus. This phenotype was observed bilaterally in 100% of all *Arvcf* ^*−/−*^ animals older than 6 months (n = 11). Interestingly, the region of opacity at 5 months appeared more deeply at 6 and 8 months (Figures 2H,J). Assuming that the opaque regions remain opaque, this observation suggests that the newly differentiating cells added to the lens during growth remained transparent. However this expanding outer region eventually acquired cloudy areas (Figures 2J,L, arrows) and became opaque by 12 months of age (Figure 2N). The nucleus also appears translucent by 12 months and by 20 months the entire lens appears completely white, has a ruptured lens capsule, and appeared to lose volume (Figure 2P). Additionally, opacities in the anterior pole were observed that overlap with suture lines by 6 months that became more prominent at 8 months and sometimes appeared as a whorl pattern (Figures 2H,J,L, Supplementary Figure S2C). To determine if the refractive ability of the lens is disturbed with the loss of Arvcf, 6 month old lenses were extracted and a Helium-Neon laser was aimed at the lens parallel to the visual axis at varying equatorial intervals (Figures 2Q,R). A composite image compiled from several individual images allowed the observation that the focal length appears longer in *Arvcf* ^*−/−*^ lenses vs. *Arvcf* ^*+/+*^ littermates (Figures 2Q,R, arrow). These focal length changes occur outside of the regions of opacity which tend to block the laser rather than refract its light path (Figure 2S). To quantify these changes, at least six lenses per genotype (*Arvcf* ^*+/+*^, *Arvcf* ^*+/-*^, and *Arvcf* ^*−/−*^) from different animals were used in the laser assay. The equatorial position of laser penetration was grouped into four equidistant regions along the lens radius and the focal length measured among laser lines from each group (Figure 2T). The average focal length was found to be significantly different when comparing *Arvcf* ^*+/+*^ and *Arvcf* ^*−/−*^ lenses only when the laser was aimed in the region between 50 and 75% of the lens radius (Figure 2U, Supplementary Figure S2F). To test whether Arvcf has any deficiencies in its mechanical properties, the dimensions of dissected lenses from control and mutant animals were measured following a mechanical load (Figures 22V–W). Upon successive placement of coverslips onto the anterior pole of the lens similar to previously published experiments (Cheng et al., 2016a), we observed that Arvcf deficient lenses from ∼60 day old animals surprisingly resisted deformation more than control animals. This was observed in both the axial and equatorial dimensions and was statistically significant when greater loads were placed upon the lenses (Figure 2X). Although the lenses appeared stiffer in the mutant animals, no significant changes in the ability of the lenses to return toward their original dimensions after the mechanical load was removed for 2 min (known as tissue resiliency) were observed (Figure 2Y). While not significantly different, the axial and equatorial dimensions of mutant lenses, on average, were closer to their preload values after removing the mechanical load (axial −3.6%; equatorial +1.2%) versus the control lenses (axial −4.8%; equatorial +2.1%). Together these data demonstrate that the loss in transparency and refractive changes is preceded by alterations to the biomechanical properties of the lens. The stiffening of tissue may be a reflection of cellular changes that occur before lens opacities in cortical cataracts.

### Disruptions in Fiber Cell Junctions and Reduced Localization of Adherens Junction Proteins Occur in the Absence of Arvcf Protein

**FIGURE 3:**
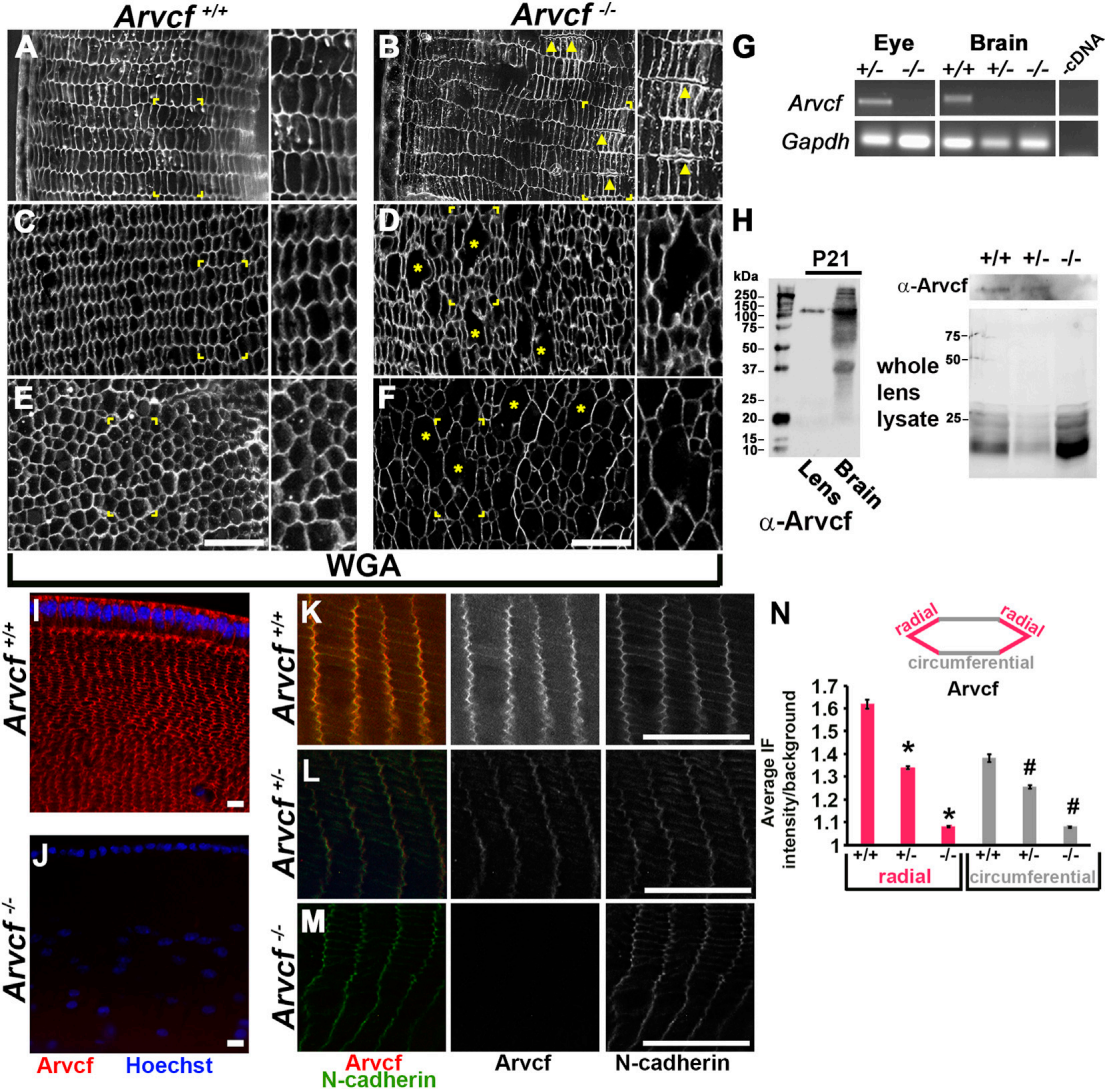
Loss of Arvcf protein leads to disrupted lens fiber cells and an increase in p120-catenin localization. **(A–F)** WGA immunolabeled (white) equatorial cryosections of 30 day old mouse lenses from animals with the indicated genotype. Magnified images of the boxed region are found to the right within each panel. **(C,D)** are images taken ∼300 microns from the lens surface and **(E,F)** are from near the lens nucleus. The yellow arrowheads point to examples of fiber cells that are often observed separated from each other in *Arvcf* ^*−/−*^ lenses and the asterisks depict regions where either the membrane is not stained or possibly missing. **(G)** RNA isolated from the eye and brain of animals with the indicated genotype was subjected to RT-PCR using primers specific to *Gapdh* and exons 3 and 4 of *Arvcf*. Exon 4 is predicted to be absent from the transcript due to the insertion of a polyadenylation signal site within intron 4–5. **(H)** An Arvcf-specific antibody was utilized on western blots from control, heterozygous, and homozygous lens and brain lysates. Left: A band near the predicted size of the longest isoform of Arvcf (105 kDa) was detected in the lens and the brain of lysates from control animals. Right: Western blots with the Arvcf antibody of lens lysates from each genotype above an image of the same gel with its total protein stained. **(I–L)** Images of equatorial cryosections of juvenile mouse lenses (P7) from *Arvcf* ^*+/+*^ (I,K), *Arvcf* ^*+/-*^ or *Arvcf* ^*−/−*^ **(L)** animals immunofluorescently co-labeled for Arvcf (red) and nuclei (blue) **(I)** or Arvcf (red) and N-cadherin (green). **(N)** Fluorescent signal intensity was measured along the junctions labeled radial or circumferential as depicted in the diagram. The graph represents the normalized mean intensity of Arvcf signal along each junction orientation. Asterisks and # symbols represent a significant difference from the control group (*p* < 0.05). A Plan-Apochromat ×40/1.3 Oil objective with wide-field fluorescent microscope was utilized. The scalebar is 25 μm.

To ascertain what cellular abnormalities may underlie these opacities, histological staining of 1 month old lenses was performed. Wheat germ agglutinin (WGA) labeling revealed that the outermost, superficial lens fiber cells of *Arvcf* ^*−/−*^ animals are similar in size and periodicity to cells from *Arvcf* ^*+/+*^ animals and are arranged in a hexagonal lattice (Figures 3A,B). However, breaks along radial fiber cell junctions are often observed in *Arvcf* ^*−/−*^ lenses (Figure 3B, arrowheads). Deeper cortical and nuclear lens fiber cells showed significantly more disruptions to the hexagonal lattice and shape of cells (Figures 3C–F). Large spaces between cell membranes are often observed in *Arvcf* ^*−/−*^ but not *Arvcf* ^*+/+*^ lenses that appear to be irregularly sized fiber cells. It is unclear whether these are due to fusion events of two or more cells or are abnormally swollen, but they do appear to be cellular and not intracellular space or histological artifacts as cytoplasmic αB-crystallin labeling is localized to these regions (Supplementary Figure S3A). Strikingly, these lenses already have signs of cellular disorganization at a time point months before cortical opacities are readily observed suggesting that these disruptions in cellular architecture are not initially a source of opacity.

The cellular disruptions and opacities are likely due to the absence of any detectable Arvcf expression in these animals. Both RT-PCR and western blotting assays were unable to detect expression of Arvcf RNA and protein from Arvcf homozygous mutant whole eyes, lenses, and brain tissue of 21 day old mice (Figures 3G,H, Supplementary Figures S3B–D). In addition, Arvcf was not detected following immunofluorescent labeling of histological sections of lenses from *Arvcf* ^*−/−*^ animals (Figures 3I–M). Arvcf antibody labeling is strong along both the shorter bicellular junctions, orientated along the equatorial radius (radial), and longer bicellular junctions, orientated parallel with the equator (circumferential), in *Arvcf* ^*+/+*^ lens fiber cells (Figure 3K). Localization of detectable protein to both junctions is reduced in *Arvcf* ^*+/-*^ lens fiber cell junctions (Figure 3L) and completely absent from those of *Arvcf* ^*−/−*^ lens fiber cells (Figure 3M). Protein expression also appears absent from *Arvcf* ^*−/−*^ embryonic lenses during development (Supplementary Figures S1G–I). Measuring the immunofluorescent intensity along the radial and circumferential fiber cell junctions from *Arvcf* ^*+/+*^ *Arvcf* ^*+/-*^ *Arvcf* ^*−/−*^ lenses revealed significant decreases in junction localization corresponding to gene dosage (Figure 3N).

**FIGURE 4:**
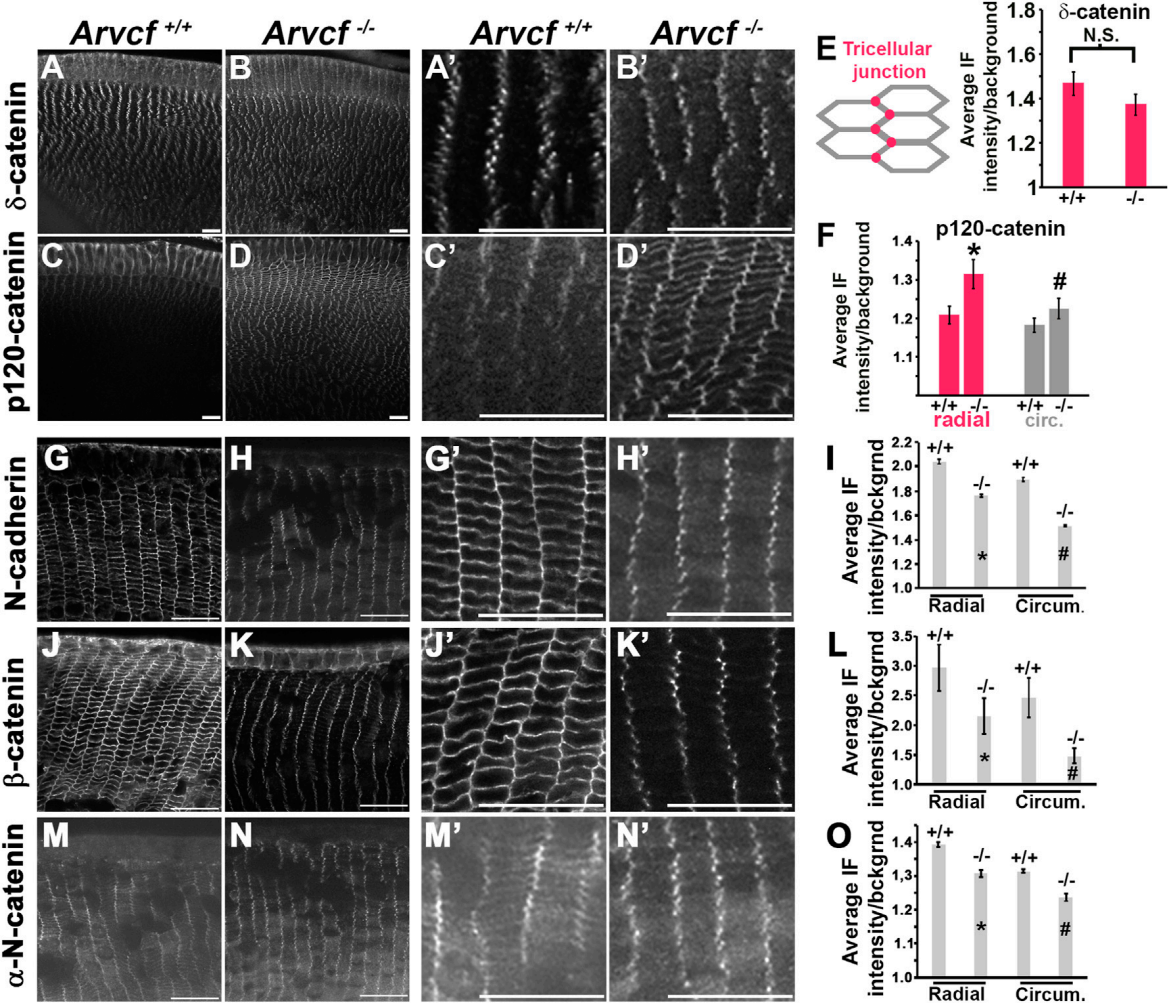
Arvcf is required for adherens junction protein membrane localization. **(A–D)** Equatorial cryosections of juvenile mouse lenses (P7) from *Arvcf* ^*+/+*^ **(A,C)** or *Arvcf* ^*−/−*^ **(B,D)** animals immunofluorescently labeled for δ-catenin **(A–B)** or p120-catenin **(C–D)**. Magnified views of the lens fiber cells within ∼50 μm of the lens surface from **(A–D)** are displayed in the panels immediately to the right. **(E–F)** The graphs represent the mean and standard error of tricellular fluorescent intensity of δ-catenin **(E)** or the bicellular junctional intensity of p120-catenin **(F)**. The asterisks/# symbols represent statistically significant differences between the control and experimental gropus (*p* < 0.05). **(G–O)** Equatorial cryosections of juvenile (P30) day old lenses from *Arvcf* ^*+/+*^ **(G,J,M)** or *Arvcf* ^*−/−*^ **(H,K,N)** mice were immunofluorescently labeled with antibodies specific for N-cadherin **(G–H)**, β-catenin **(J–K)**, and αN-catenin **(M–N)**. Magnified views of the lens fiber cells within ∼50 μm of the lens surface are depicted to the right. The graphs in **(I,L,O)** represent the mean and standard error of junctional measurements taken as shown for Figure 3N. The asterisks/# symbols represents a statistically significant difference from the control group (*p* < 0.05). A Plan-Apochromat ×40/1.3 Oil objective with wide-field fluorescent microscope was utilized. Scalebar = 25 microns.

Because of the potential for compensatory increased expression of other p120-catenin subfamily members in the absence of Arvcf, the expression of δ-catenin and p120-catenin were examined by immunofluroescent labeling mutant lenses. Within 4-week old mice, the localization of δ-catenin appears unaffected by the loss of Arvcf and maintains its tricellular localization pattern (Figures 4A,B). However, the localization of p120-catenin in both the radial and circumferential bicellular junctions appeared more intense (Figures 4C,D). This is not due to differential labeling between experiments and was observed in multiple lenses (Supplementary Figure S3E, n = 4). Upon measuring their intensities, the tricellular junction intensity of δ-catenin does not significantly vary between *Arvcf* ^*+/+*^ vs. *Arvcf* ^*−/−*^ (Figure 4E) but a significant increase in junctional p120-catenin was observed (Figure 4F). These results suggest that compensatory, feedback mechanisms may exist to regulate p120-catenin expression and/or localization but not δ-catenin in response to loss of Arvcf function.

The p120-catenin subfamily of catenins are known to bind to the juxtamembrane domain of cadherins and thought to increase the stability of adherens junctions. Histological cryosections were prepared from 4-week old animals to visualize whether a reduction in membrane localization of identified proteins in lens fiber cells is observed in the absence of Arvcf (Figure 4). N-cadherin localization to radial and circumferential junctions appears reduced, particularly along the longer, circumferential junctions (Figures 4G,H). Quantification of immunofluorescent intensity confirmed this observation (Figure 4I). Interestingly, the tricellular localization of N-cadherin appears retained (Figure 4H), possibly because δ-catenin functions similarly to Arvcf and is still present in *Arvcf* ^*−/−*^ lenses. Likewise, β-catenin (Figures 4J–L) and α-N-catenin (Figures 4M–O) are also reduced in the membranes of *Arvcf* ^*−/−*^ lens fiber cells to a significant degree and appear retained in the tricellular junctions. This result suggests their association with the adherens junctional complex in lens fiber cells is at least partially dependent on Arvcf.

### Cadherin Nanoclusters are Reduced in Size and Density in the Absence of Arvcf

**FIGURE 5:**
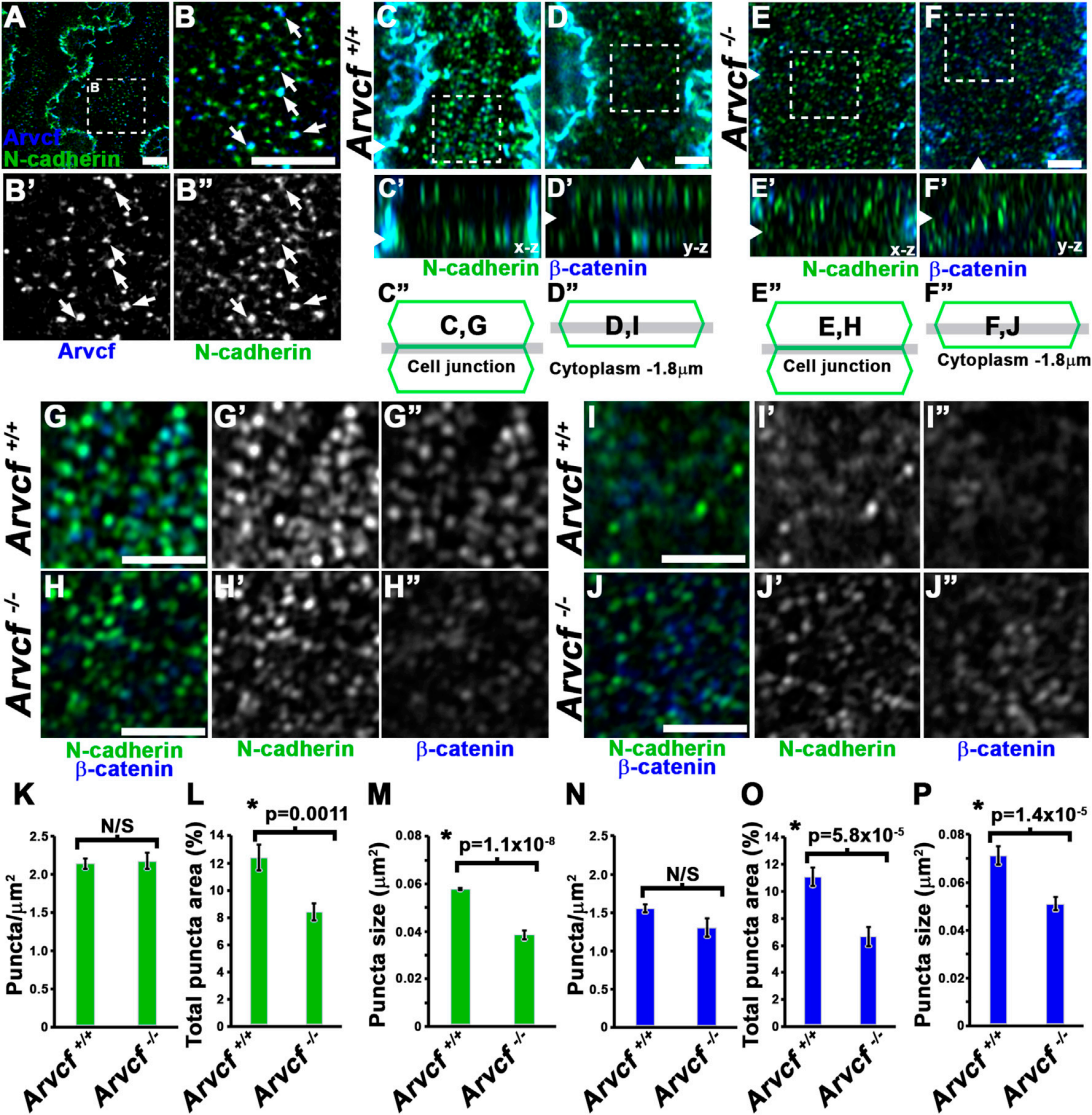
Arvcf is required for organization of N-cadherin and β-catenin in pAJs. **(A–B)** Whole lens fiber cells immunofluorescently co-labeled for Arvcf (blue) and N-cadherin (green). The squared region in A is the region magnified in panel **(B)**. The arrows represent N-cadherin nanoclusters that appear to be overlapping with Arvcf protein. Note that this is a common occurrence. **(C–F)** Whole lens fiber cells from *Arvcf* ^*+/+*^ **(C–D)** or *Arvcf* ^*−/−*^ **(E–F)** mice immunofluorescently co-labeled for β-catenin (blue) and N-cadherin (green) and imaged when focused on a z-plane within the bicellular membrane of two neighboring cells **(C,E)** or focused on a z-plane through the cytoplasm of a single fiber cell ∼1.8 μm from the membrane **(D,F)**. Note that the cytoplasmic signal is relatively low in control but greater in the mutant lens fiber cells. **(G–J)** Magnified regions outlined by the squared areas in **(C–F)** showing both the overlapping and individual signals. **(K–P)** Attributes including the density **(K,N)**, total area coverage **(L,O)**, and individual size **(M,P)** of N-cadherin **(K–M)** and β-catenin **(N–P)** puncta were quantified from immunofluorescently labeled *Arvcf* ^*+/+*^ and *Arvcf* ^*−/−*^ fiber cells and their means and standard error are depicted in the corresponding graphs. The asterisks symbols represents a statistically significant difference from the control group (*p* < 0.05). A Plan-Apochromat ×63/1.40 oil objective was utilized with a Zeiss Airyscan equipped microscope. All scalebars represent 2 microns.

The p120-catenin subfamily of catenins strengthen the cadherin complex through facilitating the clustering of cadherin monomers into structures called cadherin nanoclusters (Yap et al., 1998; Ishiyama et al., 2010; Yap et al., 2015). These nanoclusters have historically been referred to as spotty or punctate adherens junctions (pAJs) and are observed in lens fiber cells with electron microscopy (Lo, 1988; Niessen and Gottardi, 2008). Recent super-resolution, light microscopy studies have demonstrated that cadherin nanoclusters vary in size and range in diameter from approximately 50–60 nm at the smallest organizational size to as large as 1–2 microns (Yap et al., 2015). Utilizing high-resolution microscopy (Airyscan) to image the broader, circumferentially orientated junctions following immunolabeling of cortical lens fiber cells, demonstrated that Arvcf signal appears as puncta that are often in close association with N-cadherin puncta and appear approximately ∼150 nm in size (Figures 5A,B, white arrows). This supports a role for Arvcf in the stabilization of these nanocluster structures. To determine if these structures are affected by the loss of *Arvcf*, whole fiber cells dissected from the cortical region of the lens from 1 month old mice were imaged following immunofluorescent labeling with N-cadherin and β-catenin antibodies (Figures 5C–J). Upon focusing on a plane parallel to the bicellular junctions of control lens fiber cells, N-cadherin and β-catenin labeling appeared as discrete puncta along the entire lateral membrane surface (Figures 5C,G). The puncta appear reduced in intensity and are more diffuse in appearance when observed at the level of the cytoplasm where junctions are not present (Figures 5D,I), which may be indicative of endocytosed (N-cadherin) or adherens junction dissociated (β-catenin) protein. The contrast between the immunolabeling of junctional and cytoplasmic regions is also well defined in the z-stack projections (Figure C′ and D’). When imaging *Arvcf*-deficient lens fiber cells the difference between the bicellular junctional plane and cytoplasmic planes are difficult to discern from each other in either the x-y or z projection planes possibly because of destabilized cadherin leads to more cadherin endocytosis and accumulation of N-cadherin/β-catenin within the cytoplasm (Figures 5E,F,H,J). The size of the bicellular junctional puncta in Arvcf-deficient lens fiber cells also appear smaller (Figure 5E vs. Figure 5F). Quantifying the size and density of the junctional puncta of lens fiber cells from each phenotype revealed that while the density of individual puncta is not significantly different (Figures 5K,N), the area of individual N-cadherin and β-catenin puncta (Figures 5M,P) and the percentage of the total area occupied by either N-cadherin (Figure 5L) or β-catenin (Figure 5O) are significantly reduced. Utilizing the average area of puncta and assuming that the puncta are roughly circular, the calculated diameter of N-cadherin puncta are approximately ∼155 nm in diameter in control cells and are reduced to ∼124 nm in the absence of *Arvcf*. These data suggest that Arvcf is required to maintain the size of cadherin nanocluster complexes of lens fiber cell bicellular membranes and may be indicative of a reduction in cell adhesion.

### Lens Fiber Cell Morphology is Disrupted in the Absence of Arvcf due to Reduced Adherens Junction Proteins

**FIGURE 6:**
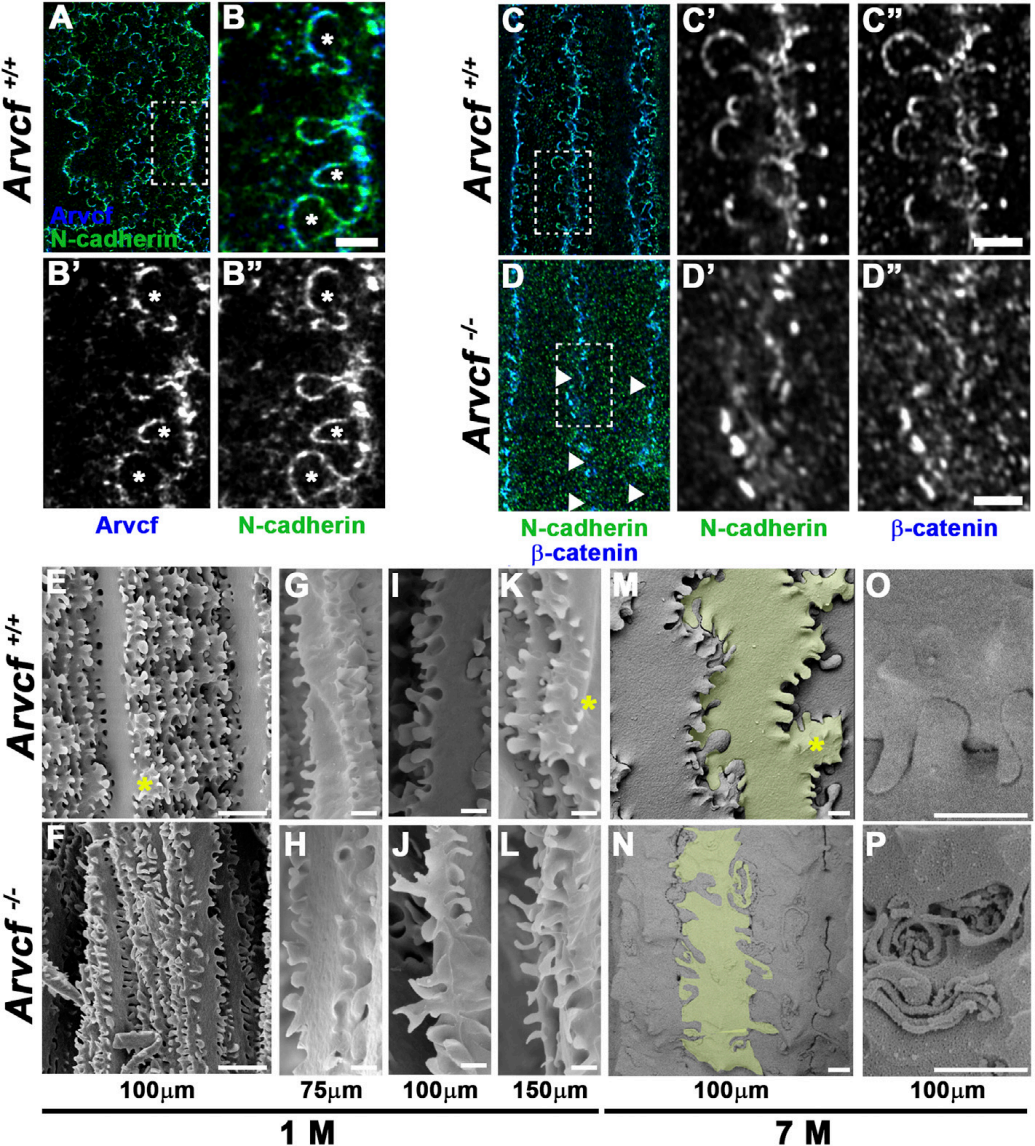
Arvcf is required for lens fiber cell interlocking protrusion morphology. **(A–B)** Whole lens fiber cells immunofluorescently co-labeled for Arvcf (blue) and N-cadherin (green). The box in A is magnified in **(B)**. The asterisks mark interlocking protrusions with lateral margins that are enriched for both Arvcf and N-cadherin. A Plan-Apochromatic CS2 ×100/1.4 oil objective was utilized with a Leica TCS SP8 STED equipped microscope. **(C–D)** Whole lens fiber cells immunofluorescently co-labeled for β-catenin (blue) and N-cadherin (green). The box in C is magnified in **(D)**. The arrowheads mark regions of discontinuous signal of both β-catenin and N-cadherin in *Arvcf* ^*−/−*^ fiber cells **(D)**. Also note that interlocking protrusions are not well defined by β-catenin and N-cadherin in *Arvcf* ^*−/−*^ fiber cells. A Plan-Apochromat ×63/1.40 oil objective was utilized with a Zeiss Airyscan equipped microscope. **(E–P)** SEM images of lens fiber cells from 1 month old **(E–N)** and 7 month old **(M–P)** *Arvcf* ^*+/+*^ and *Arvcf* ^*−/−*^ lenses located at the indicated depths from the lens surface. Note that while N-cadherin/β-catenin do not delineate interlocking protrusion borders, they are still present in abundance but are often elongated and misshapen. Asterisks mark regions where the characteristic paddle structures appear missing. Lens fiber cells in **(M–N)** are shaded to view morphology. Scalebars in **(E–F)** = 10 microns, **(G–P)** = 1 micron.

**FIGURE 7:**
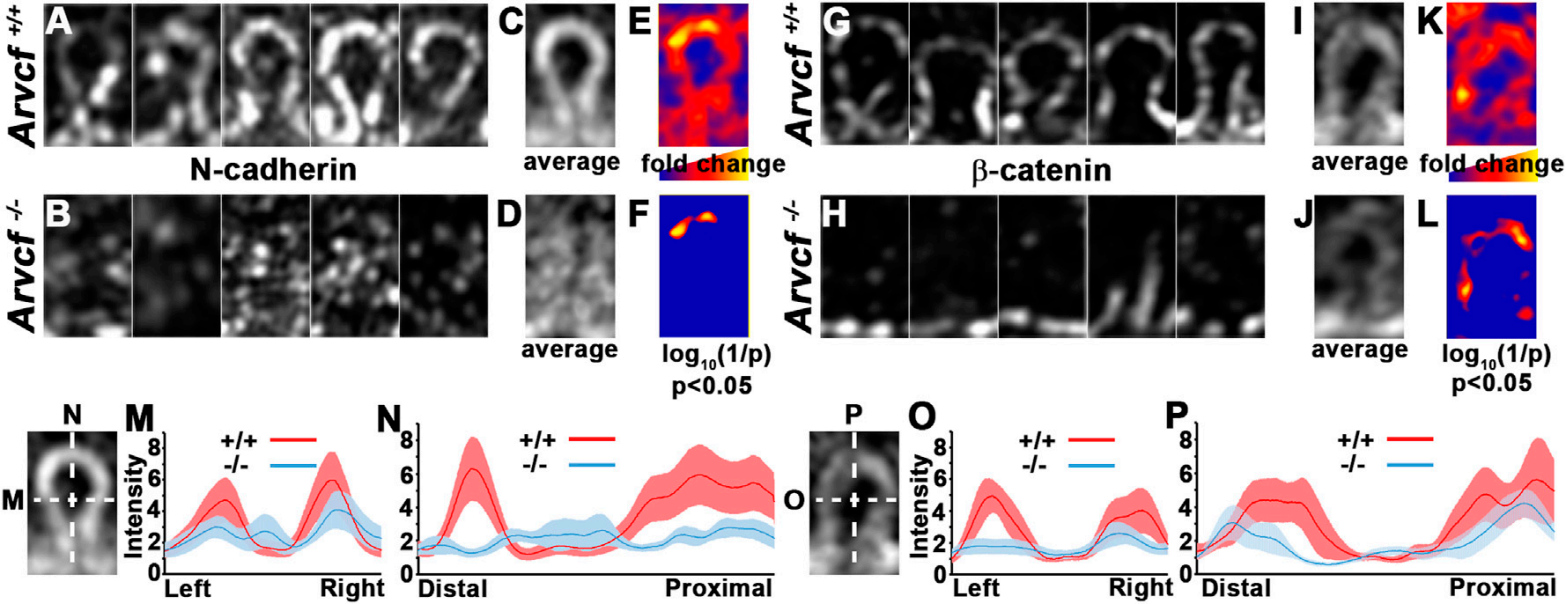
Arvcf is required for N-cadherin/β-catenin localization within interlocking protrusions. **(A–L)** Representative individual images of similarly sized, interlocking protrusions from *Arvcf* ^*+/+*^ **(A,G)** and *Arvcf* ^*−/−*^ **(B,H)** lenses immunofluorescently labeled with N-cadherin **(A–B)** or β-catenin **(G–H)** were aligned and used to measure the fluorescent intensity of each pixel. The mean intensity for each pixel was calculated and used to generate an artificial image that represents the average signal intensity for N-cadherin **(C–D)** or β-catenin **(I–J)** in *Arvcf* ^*+/+*^ **(C,I)** and *Arvcf* ^*−/−*^ **(D,J)** fiber cell interlocking protrusion. **(E,K)** represent an artificial image where the fold change at each pixel position is indicated by a heatmap. Regions where *Arvcf* ^*+/+*^ interlocking protrusions have more signal than *Arvcf* ^*−/−*^ protrusions have warmer (redder) colors and regions where differences are less pronounced have cooler colors (bluer). **(F,L)** represent an artificial image indicating where statistically significant differences in pixel intensity are located. Note that the tips of interlocking protrusions have the greatest and statistically significant differences (*p* < 0.05). **(M–P)** The mean intensity and standard error of pixels from individual interlocking protrusion images along the lines drawn parallel to the distal-proximal or left-right axes from each experimental group are depicted in the graphs. Note the depressed N-cadherin and β-catenin intensity along both axes *Arvcf* ^*−/−*^ protrusions. All images were taken with a Plan-Apochromat ×63/1.40 oil objective and a Zeiss Airyscan equipped microscope was utilized.

In addition to their localization to bicellular junctions, high-resolution imaging also revealed that Arvcf and N-cadherin are strongly localized to the base of and along the lateral sides of the interlocking protrusions (Figures 6A,B). Interestingly, the observed signal appears largely absent from the broad sides of the interlocking protrusions (Figure 6B, asterisks) suggesting that cadherin-based junctional complexes are formed solely along the protrusion margins. Strikingly, the normally intense and continuous tricellular junctional signal of N-cadherin and β-catenin near the protrusion bases of cortical lens fiber cells (Figure 6C) appears in an intermittent pattern in the absence of *Arvcf*. (Figure 6D, arrowheads). Furthermore, N-cadherin and β-catenin are rarely observed at the margins of interlocking protrusions and no longer appear to delineate their margins (Figure 6C vs. Figure 6D). Because this observation could be due to the absence of protrusions, scanning electron microscopy was performed on 1 month-old lenses from control and Arvcf deficient mice. While the Arvcf mutant lenses have an abundance of interlocking protrusions that is similar to control lenses in the absence of Arvcf (Figure 6E vs. Figure 6F), they are often misshapen and elongated in several regions of varying depths (Figures 6G,I,K vs. Figures 6H,J,L and Supplementary Figures S4A,B). Furthermore, well-defined paddle structures are difficult to distinguish unlike in control lenses (Figures 6E,K,M, asterisks). Older, cataractous lenses display an exacerbated interlocking protrusion phenotype and possess extremely long interlocking protrusions that curl up within the spaces between fiber cells (Figures 6M–O vs. Figures 6N–P). Therefore, the inability of N-cadherin/β-catenin to define the protrusion boundaries is not due to their absence but instead due to their extreme mislocalization within them. To characterize this mislocalization, super-resolution images of individual protrusions immunofluorescently labeled with N-cadherin or β-catenin were collected and processed for comparison between genotypes (Figures 7A,B,G,H). Because individual protrusions from Arvcf deficient lens fiber cells are difficult to identify from N-cadherin or β-catenin localization alone (Figures 7B,H), co-labeling with Aquaporin-0 was utilized to locate them (Supplementary Figure S4C). The fluorescent intensity of every pixel from 10 to 16 aligned, morphologically similar individual interlocking protrusion images was quantified and compared with the pixels at the same position of other images (Supplementary Figure S4C). The average pixel intensity at each position was quantified and used to generate an “average image” for each genotype (Figures 7C,D,I,J). When the fold change at each pixel was determined and represented as a heat map image it indicated that changes were greatest along the borders of the protrusions (Figures 7E,K). Statistically significant differences were also represented as a heat map and found at the protrusion apices (Figures 7F,L) and along the lateral boarder (Figure 7L). Differences in fluorescent intensity are also visualized with more conventional line graphs (Figures 7M–P). Together these data demonstrate that N-cadherin and β-catenin require Arvcf to localize to the margins of interlocking protrusions.

## Discussion

### Arvcf Deficient Lenses are a Model for Age-dependent Cortical Cataracts

The development of age-related cortical cataracts and loss of cadherin-associated proteins in fiber cell membranes observed in the absence of Arvcf is a significant finding because it demonstrates that this type of cataract can be caused by a reduction in adherens junction mediated adhesion. This information has not yet come to light likely because loss of function analyses in mice focused on proteins with adhesive function have severe lens defects or die prematurely. Conditional ablation of N-cadherin, the dominant cadherin in lens fiber cells, disrupts secondary lens fiber cell elongation leading to a severely disorganized lens (Pontoriero et al., 2009; Logan CM. et al., 2017; Logan C. M. et al., 2017). Lens-specific removal of β-catenin similarly causes significant disruptions to lens fiber cells during development (Cain et al., 2008). It is possible that lens specific removal of other genes encoding cadherin-complex proteins expressed in the lens fibers such as α-N-catenin, p120-catenin, δ-catenin and/or afadin may also cause age-related cortical cataracts however these experiments have yet to be reported (Jun et al., 2012; Lachke et al., 2012; Lang et al., 2014; Maddala and Rao, 2017). Aquaporin-0 and Connexin 50 deficient mice also have lens fiber cell adhesive function, but the cataracts that occur in these models are nuclear, present at an early age, and are likely related to ion and water transport functions of the proteins (White et al., 1998; Shiels et al., 2001; Kumari et al., 2013; Hu et al., 2017; Gu et al., 2019; Varadaraj and Kumari, 2019). Thus, the Arvcf mutant model is ideal for determining what occurs downstream of cell adhesion failure to cause opacities and will be the subject of future investigation.

### p120-Catenin May Genetically Compensate for Arvcf

Arvcf does not appear to be a required component of adherens junctions during secondary lens fiber cell differentiation at the embryonic or even at young adult stages due to the absence of an embryonic phenotype and transparency of the lens within superficial fiber cells. This could be due to genetic compensation from p120-catenin, which is the paralog with the greatest sequence similarity to Arvcf and is expressed in primary and secondary lens fiber cells during development (data not shown) (Pieters et al., 2012). Because p120-catenin and Arvcf function similarly, p120-catenin is likely sufficient for N-cadherin retention within adherens junctions during development. The upregulation of p120-catenin in fiber cells in the absence of Arvcf may also suggest why opacity occurs in a specific region (∼100–150 microns from the surface). The outermost cells where p120-catenin is highest may have sufficient stabilization of the cadherin complex to maintain adhesive contact and transparency. Because the p120-catenin upregulation appears restricted to only the cortical region of the lens, it does not explain why the central lens remains transparent. The development of opacities may be related to age-related changes in lens fiber cell mechanics where interfaces between the stiffer, more central regions and comparatively softer lens cortex may be subject natural breaks or slippage between regions as has been previously proposed (Fisher, 1973; Pau, 2006; Michael et al., 2008). The hydrostatic pressure gradient between central and more peripheral lens fiber cells may also contribute to mechanical environment differences and influence where opacity occurs (Gao et al., 2011).

### Requirement for Arvcf in the Organization of Punctate Adherens Junctions in Juvenile Lens Fiber Cells

The use of super-resolution fluorescent microscopy has also enabled this study to probe the consequences of Arvcf deficiency on pAJ organization in the junctions juvenile lens fiber cells. With this technique we observed that the N-cadherin/Arvcf-residing adherens junctions are organized in clusters that are on the order of ∼150 nm, making them smaller than and distinct from larger microclusters found in places such as the apical adherens junctional complex (Yap et al., 2015). Because Airyscan imaging has a lower end resolution capability of ∼140 nm, it is possible that these structures are a concentration of smaller order nanoclusters which can be as small as 50–60 nm in size (Quang et al., 2013; Huff, 2015; Wu et al., 2015). Thus, the smaller structures we observe could be due to fewer nanocluster subunits that fail to congregate together or that they are simply smaller whole nanocluster subunits that consist of fewer N-cadherin/β-catenin molecules. Nanoclusters of cadherin molecules can form in the absence of adhesion and it remains possible that some of these structures we observe are non-adherent (Wu et al., 2015). However transmission electron microscopy (TEM) has revealed that the spotty/pAJ of lens fiber cells from several species appear to be approximately ∼100–200 nm in size suggesting that these structures are indeed adherent (Lo, 1988; Lo et al., 1997). These data also support the possibility that the smaller nanoclusters we observe are likely due to smaller adhesion sites rather than fewer smaller nanoclusters. While the absence of Arvcf appears to diminish the size of these nanoclusters it is important to note that they are still present. Given that the p120-catenin can stabilize cadherins by binding to the juxtamembrane domain and masking the endocytosis signal, it is unclear why these cadherin nanoclusters persist in the absence of homologous Arvcf (Xiao et al., 2005; Miyashita and Ozawa, 2007). It is possible that Arvcf is necessary for some but not all N-cadherin retention and that additional proteins that have previously been implicated in protecting N-cadherin from endocytosis, such as β-catenin or NMDAR, can serve this role (Tai et al., 2007; Chen and Tai, 2017).

### Arvcf Dependent Morphology of Lens Fiber Cell Interlocking Protrusions

Another consequence of Arvcf deficiency is the disruption of N-cadherin and β-catenin localization within interlocking protrusions of lens fiber cells. Along with distortions of their shape and the abundance of Arvcf protein normally observed suggest that Arvcf stabilization of adherens junctions is integral to their function. These protrusions likely prevent fiber cells from sliding past each other via their interlocking nature but also likely provide stability through the creation of a large amount of surface area for adherens junction protein complexes to reside as well. Our observations of N-cadherin within the lateral domains of interlocking protrusions stands in contrast to previously published data where it appeared absent (Cheng et al., 2016b). However, we observe a similar absence when individual lens fiber cells have been pulled from their neighbors and imaged individually (Supplementary Figure S4) and it is likely that the cadherin complexes can be pulled out of the membrane when dissected in this manner. While this occurs most of the time, we have observed paired N-cadherin localization along the protrusion membrane on occasion when fiber cells become separated from each other (Supplementary Figure S4). Furthermore, pAJ-like structures have also been observed via TEM between interlocking protrusions and the pocket of neighboring cells supporting the presence of N-cadherin (and Arvcf) containing adherens junctional complexes in protrusion membranes (Biswas et al., 2010; Biswas et al., 2016). Adherens junction complex formation within these structures may also be important for stabilizing the membrane to allow optimal function for aquaporin-mediated water transport. Because water/ion movements are extremely important for lens physiological mechanisms that protect it from oxidative damage and preserve transparency it is possible that Arvcf deficiency may result in cortical cataracts via a disruption to these protective mechanisms.

The lack of adhesion complexes within interlocking protrusions may also be the reason for the disrupted organization and shape of deeper cortical lens fiber cells. The cellular separations we observed along the radial junctions may be easily explained as a direct result of reduced adhesion and therefore could be more prone to separation. The radial regions may be particularly sensitive to this because it is where the interlocking protrusions are concentrated. The abnormal elongation of interlocking protrusions could also be a direct effect of reduced adhesion. These lens structures have been morphologically and molecularly compared to dendritic spines of neurons (Frederikse et al., 2012) Reduced N-cadherin or α-N-catenin function cause spine elongation due to reduced adhesion (Togashi et al., 2002; Abe et al., 2004; Xie et al., 2008). Therefore loss of Arvcf in lens fiber cells may mimic what occurs in the absence of adhesion in neurons and cause remodeling of the protrusion structures. Part of this mechanisms may include the ability of members of the p120-catenin subfamily to regulate Rho-GTPases and actin dynamics in many contexts including during dendritic spine morphogenesis (Elia et al., 2006; Arikkath et al., 2009). Elongation of interlocking protrusions in lens fiber cells could therefore be due to an increase in actin polymerization that is dependent or independent of reduced adhesion. Interestingly, these elongation phenotypes, as well as the presence of swollen fiber cells are similar to what is observed in Aquaporin-0 knock out mice (Lo et al., 2014). The shared phenotypes could be due to Aquaporin-0’s adhesive or water transport functions and determining whether Aquaporin-0 function is related to Arvcf is a worthy avenue of investigation.

### Arvcf-dependent Biomechanical Properties of the Lens

Although adhesion is likely reduced between lens fiber cells of Arvcf deficient lenses, it is somewhat surprising that they are stiffer. It is reasonable to speculate that if fiber cells have a reduction in the protein complexes that adhere cells to one another then the tissue may be predisposed to deform more. However, the increased stiffness observed here in lenses from 60-day old mice demonstrates that Arvcf-dependent adhesion alone is not a major contributor to lens tissue stiffness at this age. Similar to our results, lenses from 2.5 months- to 4 month-old aquaporin-0 deficient mice have an increased capacity to resist deformation (Kumari et al., 2015; Gu et al., 2019). It is possible that the increase in stiffness for both models indicate a shared mechanism, especially given the observations from this study and others (Logan C. M. et al., 2017; Maddala and Rao, 2017). Alternatively, increased stiffness in Arvcf deficient lenses may also be related to the Rho-GTPase regulatory function of the p120-catenin subfamily (Fang et al., 2004; Anastasiadis, 2007). Loss of Arvcf may cause disrupt Rho-GTPase-dependent actin-cytoskeleton regulation and cause a stabilizing network of F-actin to polymerize. Intermediate filament and tropomodulin knock-out mice have lenses with decreased stiffness (Fudge et al., 2011; Gokhin et al., 2012) making it plausible that altering the actin cytoskeleton may result in tissue stiffening. Identifying the underlying mechanisms behind why the biomechanics of the lens is altered in this mouse model may ultimately facilitate an understanding of the etiology of age-related cortical cataracts.

Together, these data demonstrate that Arvcf is required to maintain transparency with age through the stabilization of the N-cadherin containing adherens junctions between lens fiber cells. By serving as a model of age-dependent cortical cataracts, further analysis of Arvcf-deficient mice has the potential to provide new information on the development of cortical cataracts with age. Furthermore, this study may indicate that the stability of the adherens junctional complex could be a biomarker of the aging process that predisposes it for cataract formation. It is speculated that loss/reduction of adherens junctions between fiber cells may induce opacities by disrupting ion and water transport, the architecture of the cytoskeleton, and/or the biomechanical properties of the lens.

## Materials and Methods

### Arvcf Mouse Model Generation and Maintenance

The *Arvcf* targeted mouse line, *Arvcf* ^*tm1e(EUCOMM)*^Wtsi, was purchased from the European Mouse Model Archive (EMMA ID 04548). The line was generated from the insertion of a lacZ/Neomycin cassette (L1L2_gt0) to generate an ES cell clone (EUCOMM EPD0102_1_D06) which targeted the *Arvcf* locus on chromosome 16 at position 18396535 between exons 3 and 4 of the longest Arvcf transcript (Supplementary Figure S1A). The 3′ loxP site was not preserved during targeting preventing this allele from having conditional potential. Genotyping was performed utilizing the cassette flanking primers 5′-GCT​GAC​CTA​ACC​ATG​GTT​ACG (for) and 5′-CAA​GAC​AAG​TCC​ATC​TGG​ACC (rev) and the targeting cassette residing primer 5′CAA​CGG​GTT​CTT​CTG​TTA​GTC​C (p3) to yield a 564 nucleotide and/or a 430 nucleotide band in a 1% agarose gel with PCR using Dream Taq (Thermofisher) and the following protocol: 95°C for 5 min (94°C for 30 s, 65°C for 45 s, 72°C for 45 s, 35cycles) 72°C for 60 s (Supplementary Figure S1B).

### Laser Refraction Experiments

The refraction chamber was made from a clear acrylic box with a ¾” hole drilled in its side and a 1” disc of optical glass (Thorlabs: WG11050) glued to the outside. The mouse lenses were placed upon a pedestal made from a glass cloning cylinder and a piece of plastic glued to the top creating a small hole for the lens to rest on its equatorial side. A 0.8 mW, 632.8 nm helium/neon laser (Thorlabs: HNLS008L) was mounted on a vertically articulating platform and aimed through a Plano Convex Lens (Thorlabs: LA1131) which focused the beam parallel to the light axis of the mouse lens tissue. The chamber was flooded with phosphate-buffered saline (PBS) mixed with a small amount of powdered milk to increase the turbidity of the solution. Images of no fewer than 6 lenses dissected from at least 5 different animals and the surrounding media were taken following incremental raising (13–17 increments) of the platform-mounted laser such that the beam could be imaged across the equatorial plane of the lens. Measurements of focal lengths and the position of the beam within the lens were performed using Zen software (Zeiss). ANOVA analysis was used to determine whether statistical differences were observed between genotypes followed by post-hoc t-tests.

### RT-PCR and Western Blot Analysis

RNA was extracted from whole eyes and cerebellar brain tissue using commercially available kits (RNeasy/Qiagen) and used to prepare cDNA (Verso cDNA synthesis kit/Thermo Fisher). PCR was performed utilizing *Arvcf {*5′-GCT​GCT​GGC​ACC​CTG​GTC​AT (for) and 5′-GTC​TCA​GTC​CGC​CGG​GTT​GTA (rev)} and *Gapdh* {5′-CAT​CAC​TGC​CAC​CCA​GAA​GAC​TG (for) and 5′-ATG​CCA​GTG​AGC​TTC​CCG​TTC​AG (rev)} specific primers, Dream Taq (Thermofisher) and the following protocol: 95°C for 5 min (94°C for 30 s, 60°C for 60 s, 72°C for 30 s, 39 cycles) 72°C for 60 s to yield a 364 (*Arvcf*) or 110 (*Gapdh*) basepair band in a 1% agarose gel. Western blotting of lens or brain lysates was performed by extracting protein from two lenses or 1 cerebellar hemisphere per experimental group by first homogenizing the tissue in 1 × PBS pH 7.0 100 mM EGTA with an electric handheld homogenizer for 2 min, centrifuged (30 min 17000 × g at 4°C) and the pellet washed in homogenization buffer with 50 mM DTT and recentrifuged for (15 min 17000 × g at 4°C). RIPA buffer (200 μl/Pierce) mixed with 2 μl of protease inhibitors (HALT protease inhibitor cocktail/Thermo Fisher) was used to resuspend the pellet and incubated for 45 min at 4°C with gentle agitation. The lysate was centrifuged again (15 min 17000 × g at 4°C) and the lysate used in SDS-PAGE analysis using 12% precast TGX Stain-Free polyacrylamide gels (Bio Rad) which allows for total protein in the gel and/or blot to be visualized with UV light and is more reliable than antibody-based housekeeping gene loading controls (Rivero-Gutierrez et al., 2014). Arvcf (Bethyl Laboratories, A303-310A) and β-catenin specific antibodies (1:500, BD biosciences, BDB610153) were utilized at 1:1,000 dilution on blots and detected with HRP conjugated secondary antibodies, Clarity ECL (Bio Rad) reagent, and a ChemiDoc imager (Bio Rad).

### Immunofluorescent Labeling and Imaging

Mouse embryo and lens tissue was fixed in 4% (embryos, whole lenses) or 2% (dissected cortical lens fiber cells) paraformaldehyde. For histological sections, tissue was embedded in OCT medium (Tissue-Tek) following 15 and 30% sucrose infiltration and a cryostat used to generate 10–20 μm sections. Dissected cortical lens fiber cells were prepared in a manner similar previously published methods (Cheng et al., 2016b). The staining protocols used were similar to those previously described (Houssin et al., 2020). The following primary antibodies were utilized followed by a combination of Alexa Fluor secondary antibodies conjugated with 488, 568, 594, or 647 fluorophores (Invitrogen). Antibodies: Arvcf (1:500 for sections; 1:250 for whole lens fiber cells, Thermofisher, PA5-64129), N-cadherin (1:500 BD Biosciences, 610,921), αN-catenin (1:250, ABclonal A15269), δ-catenin (1:500, Thermofisher, PA5-53275), p120-catenin (1:200, BD Biosciences, BDB610133), β-catenin (1:500, BD biosciences, BDB610153), β-catenin (1:200, GeneTex, GTX101435), Aquaporin-0 (1:200, Alpha Diagnostics International, AQP01-A), αB-crystallin (1:500, DSHB, CPTC-CRYAB-3). Fluorescently labeled Hoechst 33,342 (1:1,000; Sigma, B-2261) or wheat germ agglutinin (1:200, Life Technologies, W32464/W11262) were also utilized to label nuclei or fiber cell membranes (respectively). Glass-slide mounted samples were coverslipped with Fluoro-gel medium (Electron Microscopy Sciences) and imaged with a Zeiss Axio observer inverted microscope equipped with a fluorescent light source and ×40 Plan-Apochromat objective or equipped with an LSM900 Airyscan 2 super-resolution confocal system and ×63 Plan Apochromat objective.

### Nanocluster Analysis

20 4 × 4 μm regions were extracted from images taken from an Airyscan equipped confocal microscope with a plan-apochromat ×63/1.40 oil immersion objective focused within the plane of the lateral membrane of lens fiber cells co-immunolabeled for N-cadherin and β-catenin. Images of fiber cells from at least three different lenses were used. Utilizing ImageJ, each region underwent autothresholding with the “moments” algorithm followed by using the analyze particles function to measure nanocluster attributes. Two-tailed, homoscedastic t-tests were used to determine statistical significance. The area of a circle formula was used to derive the approximate diameter of individual spots using the mean area of fluorescent signal.

### Quantitation of Immunofluorescent Signal

For all quantitative measurements, the conditions used for image acquisition were carefully selected to ensure a lack of pixel saturation and uniformity among experimental groups.

#### Cryosections

Radial and circumferential fluorescent intensity measurements was performed with ImageJ by tracing a line on top of the radial junctions and determining the mean intensity of pixels along the line. The location of the circumferential junctions were inferred from the geometry of the radial junctions and a straight line was drawn between radial points in the approximate location. While the circumferential junctional can be wavy, when a line is drawn between radial points it captures most of the signal and is not significantly different than manually tracing (data not shown). This was performed in order to overcome the lack of signal that rises above background which is sometimes observed upon IF staining of Arvcf^*−/−*^ sections. Tricellular junctional signal was measured using the ellipse tool in ImageJ that encircled a region limited to the tricellular membrane. Values were subjected to normalization by calculating the ratio with non-specific signal for each image. Measurements were made from sections from at least 3 images from at least 3 distinct animals. Images were taken 10–50 μm from the lens surface and a range of 500–700 measurements was made for each experimental group (from 50 to 60 cells/image). Two-tailed, homoscedastic t-tests were used to determine statistical significance.

#### Whole Lens Fiber Cells

Cropped regions of individual protrusions of similar size were extracted from larger Airyscan images such that the central, distal tip of signal within protrusions were at the same position. Due to disorganized signal of N-cadherin/β-catenin, protrusions of *Arvcf* ^*−/−*^ protrusions were co-labeled with an aquaporin-0 antibody which delineated their position (Supplementary Figure S4C). Fluorescent intensity of each pixel from the extracted regions was determined with the image to text function in ImageJ and compared with the pixels at the same x-y coordinate of the other images using Microsoft Excel. Once the mean intensity for each pixel was determined images were generated by importing a text file of the means using the import text image tool. The same procedure was used to generate an image using a text file containing the fold change between genotypes or a modified *p*-value from t-tests. The *p*-value was converted to a logarithmic value using the formula (Log_10_ (1/*p*-value)) Therefore, the smaller the *p*-value, the greater the pixel intensity. Intensity values of 0 were assigned to pixels that were not less than 0.05.

### Scanning Electron Microscopy

Lenses were fixed in 2.5% glutaraldehyde/0.1 M PBS pH7.4, bisected in half, incubated at 4C for 72 h, rinsed, dehydrated with a series of increasing EtOH concentrations, and dried with a critical point drier. The samples were mounted onto double-sided carbon tape on SEM stubs and underwent gold/palladium sputter coating. Samples were examined using 5 kV with a FEI *Nova* NanoSEM instrument.

### Lens Compression Tests

Lens biomechanics were tested using procedures similar to those previously described (Cheng et al., 2016a). In brief, a 45^o^ angled mirror was used in combination with an acrylic chamber filled with PBS and stereomicroscope to view an immobilized mouse lens from the equatorial side as glass coverslips were placed on the anterior side. Images were taken 45 s following the addition of each successive coverslips (10 maximum). The axial and equatorial widths were measured with Zen 2.0 and the mean strain ((*ε* = measured length-original length)/original length) was determined for each mechanical load. 8-9 lenses from at least 4 different animals were utilized per genotype. Two-tailed, homoscedastic t-tests determined statistical significance. Axial and equatorial length measurements were taken of a subset of compressed lenses (n = 5–8) 2 min following the removal of compressive load to determine lens resilience. The percent difference between the pre-load and post-load measurements ((post-load-pre-load)/pre-load) were reported.

## Data Availability

The raw data supporting the conclusions of this article will be made available by the authors, without undue reservation.

## Supplementary Material

Supplementary Figure S1 (**A)** Diagram of the elements inserted into the Arvcf locus as a result of gene targeting. The gray boxes indicate the exons and the blue half arrows indicate the approximate location of genotyping primers used to identify the targeted allele. **(B)** Representative results from genotyping following PCR amplification. **(C)** The table represents the number of embryos with the indicated genotypes collected from timed matings of heterozygous crossings. **(D–I)** Imunofluorescent labeling of F-actin and nuclei **(D–F)** or Arvcf and nuclei **(G–I)** of transverse cryosections lenses from embryos with the indicated genotype. Note that no obvious ocular phenotype is observed in Arvcf null embryos despite the absence of detectable protein. Scale bars = 50microns.

Supplementary Figure S2(A–B) Images of lenses dissected from control **(A)** or Arvcf null lenses **(B–C)**. The arrowheads demonstrate regions of cortical spoking. The white lines in B represent a transparent region that is superficial to the opaque region. **(C)** A higher magnification of an Arvcf−/− lens with a disruption at the anterior pole that occurs along a suture line. **(D–E)** Examples of lenses at the indicated ages dissected from control or Arvcf−/− animals that have been placed on an electron microscopy grid **(D)** or an ink-printer generated grid **(E)**. **(F)** Box and whisker plots that describe the quantitative data depicted in Figure 2. The line within each box represents the median value, the boxes span the first and third quartiles, the x symbol represents the mean, the whiskers represent the range of all values not considered outliers, and the circles represent the outlier values that are outside 1.5 times the interquartile range.

Supplementary Figure S3(A) A histological equatorial cryosection of a control and Arvcf-deficient lens was immunolabeled for αB-crystallin and WGA. The image is focused on a region near the lens nucleus where significant disruptions in fiber cell occur. The asterisks mark unusually large cells that are bounded by membrane (red) but filled with crystallin-containing cytoplasm (green). Note that this indicates that these regions are not intercellular breaks but rather abnormally large regions of cytoplasm. **(B)** The entire polyacrylamide gel following SDS-PAGE of lysates from control and Arvcf mutant lens lysates labeled for total protein lysate found in Figure 3H is on the left. On the right is the entire western blot of the polyacrylamide gel depicted to the left that has been immunolabeled for Arvcf protein. **(C–D)** Examples of additional western blots demonstrating the reduction of Arvcf protein in *Arvcf* ^*−/−*^ lens lysates. The white hatched boxes in D are magnified in panel **(C) (E)** Equatorial histological sections of control and Arvcf mutant lens fiber cells found within 50microns of the surface co-immunolabeled with p120-catenin (green) and β-catenin (red). Note that p120-catenin is not strongly localized to lens fiber cell membranes in control lenses but is more apparent in the lens fiber cell membranes of mutant lenses. **(F–J)** Box and whisker plots that describe the quantitative data depicted in Figures 4, 5. The line within each box represents the median value, the boxes span the first and third quartiles, the x symbol represents the mean, the whiskers represent the range of all values not considered outliers, and the circles represent the outlier values that are outside 1.5 times the interquartile range. Scale bars = 10 microns.

Supplementary Figure S4(A–B) Lower and higher magnification SEM images of a whole bisected lens or a small region of the lens approximately 100 microns from the surface from control **(A)** or Arvcf deficient lenses **(B)**. In panels A′ and B′ a single lens fiber cell was pseudocolored green to visualize their elongated nature. The scale bars in A and B = 300 microns and the scale bars in A′ and B’ = 5 microns. **(C)** Image of an individual interlocking protrusion from an *Arvcf* ^*−/−*^ lens immunofluorescently co-labeled with antibodies specific for β-catenin (green C”) or aquaporin-0 (blue C′). The diagram to the right indicates the strategy for comparing immunofluorescent images of individual protrusions. The numbers represent individual pixels labeled by position. The pixels at the same location (those that have the same position number) were compared among each protrusion image by quantifying the mean. These means were used to generate the average image in Figures 7C,D,I,J and used to perform the subsequent statistical analyses. **(D)** A single wild-type lens fiber cell immunofluorescently labeled with an N-cadherin and aquaporin-0 antibody. Note that this lens fiber cell was prepared by pulling individual fiber cells apart. **(E)** Magnified images of the regions indicated by boxes in panel **(D)**. Yellow asterisks mark cellular protrusion outlines that lie on the surface of lens fiber cells. These outlines are well delineated by both N-cadherin and Aqp0 localization and are thought to correspond to the “pocket” of an interlocking protrusion from a neighboring cell. Note that these are typically outlined well and are unaffected by dissection. This stands in contrast to the protrusions that extend into space following manual dissection (arrowheads). These are readily visualized by Aqp0 localization but not N-cadherin. **(F)** A group of wild-type lens fiber cells immunofluorescently labeled for Arvcf and N-cadherin were imaged without dissecting them from each other but have a slight separation. The yellow boxed regions are magnified in the small panels to the right. Note that N-cadherin appears to have a similar localization pattern to their neighboring cells along the margins of paddle regions. This also apparent in interlocking protrusions (bottom right panel, asterisks). Scalebars in **(D–F)** = 2000 nm.

## References

[B1] Abe K. Chisaka O. van Roy F. Takeichi M. (2004). Stability of Dendritic Spines and Synaptic Contacts Is Controlled by αN-catenin. Nat. Neurosci. 7, 357–363. 10.1038/nn1212 15034585

[B2] Abel H.-P. OʼLeary D. J. (1997). Optometric Findings in Velocardiofacial Syndrome. Optometry Vis. Sci. 74, 1007–1010. 10.1097/00006324-199712000-00021 9423991

[B3] Allegrini D. Penco S. Pece A. Autelitano A. Montesano G. Paci S. (2017). Cataract and optic disk drusen in a patient with glycogenosis and di George syndrome: clinical and molecular report. BMC Ophthalmol. 17, 107. 10.1186/s12886-017-0499-y 28659124 PMC5490087

[B4] Anastasiadis P. Z. (2007). p120-ctn: A Nexus for Contextual Signaling via Rho GTPases. Biochimica Biophysica Acta (BBA) - Mol. Cell. Res. 1773, 34–46. 10.1016/j.bbamcr.2006.08.040 17028013

[B5] Anastasiadis P. Z. Reynolds A. B. (2001). Regulation of Rho GTPases by P120-Catenin. Curr. Opin. Cell. Biol. 13, 604–610. 10.1016/s0955-0674(00)00258-1 11544030

[B6] Arikkath J. Peng I.-F. Gie Ng Y. Israely I. Liu X. Ullian E. M. (2009). -Catenin Regulates Spine and Synapse Morphogenesis and Function in Hippocampal Neurons during Development. J. Neurosci. 29, 5435–5442. 10.1523/jneurosci.0835-09.2009 19403811 PMC2763482

[B8] Bagchi M. Katar M. Lewis J. Maisel H. (2002). Associated Proteins of Lens Adherens Junction. J. Cell. Biochem. 86, 700–703. 10.1002/jcb.10258 12210736

[B9] Bassnett S. Shi Y. Vrensen G. F. J. M. (2011). Biological Glass: Structural Determinants of Eye Lens Transparency. Phil. Trans. R. Soc. B 366, 1250–1264. 10.1098/rstb.2010.0302 21402584 PMC3061108

[B10] Beebe D. C. Holekamp N. M. Shui Y.-B. (2010). Oxidative Damage and the Prevention of Age-Related Cataracts. Ophthalmic Res. 44, 155–165. 10.1159/000316481 20829639 PMC2952186

[B11] Beemer F. A. de Nef J. J. E. M. Delleman J. W. Bleeker-Wagemakers E. M. Shprintzen R. J. Opitz J. M. (1986). Additional Eye Findings in a Girl with the Velo-Cardio-Facial Syndrome. Am. J. Med. Genet. 24, 541–542. 10.1002/ajmg.1320240319 3089013

[B12] Berthoud V. M. Minogue P. J. Osmolak P. Snabb J. I. Beyer E. C. (2014). Roles and Regulation of Lens Epithelial Cell Connexins. Febs Lett. 588, 1297–1303. 10.1016/j.febslet.2013.12.024 24434541 PMC3992928

[B13] Biswas S. K. Lee J. E. Brako L. Jiang J. X. Lo W. K. (2010). Gap Junctions Are Selectively Associated with Interlocking Ball-And-Sockets but Not Protrusions in the Lens. Mol. Vis. 16, 2328–2341. 21139982 PMC2994765

[B14] Biswas S. Son A. Yu Q. Zhou R. Lo W.-K. (2016). Breakdown of Interlocking Domains May Contribute to Formation of Membranous Globules and Lens Opacity in Ephrin-A5−/− Mice. Exp. Eye Res. 145, 130–139. 10.1016/j.exer.2015.11.017 26643403 PMC4842153

[B15] Blankenship T. Bradshaw L. Shibata B. Fitzgerald P. (2007). Structural Specializations Emerging Late in Mouse Lens Fiber Cell Differentiation. Investig. Ophthalmol. Vis. Sci. 48, 3269–3276. 10.1167/iovs.07-0109 17591898

[B16] Brown N. P. Harris M. L. Shun-Shin G. A. Vrensen G. F. J. M. Willekens B. Bron A. J. (1993). Is Cortical Spoke Cataract Due to Lens Fibre Breaks? the Relationship between Fibre Folds, Fibre Breaks, Waterclefts and Spoke Cataract. Eye 7, 672–679. 10.1038/eye.1993.154 8287992

[B17] Cain S. Martinez G. Kokkinos M. I. Turner K. Richardson R. J. Abud H. E. (2008). Differential Requirement for β-catenin in Epithelial and Fiber Cells during Lens Development. Dev. Biol. 321, 420–433. 10.1016/j.ydbio.2008.07.002 18652817

[B18] Casteels I. Casaer P. Gewillig M. Swillen A. Devriendt K. (2008). Ocular Findings in Children with a Microdeletion in Chromosome 22q11.2. Eur. J. Pediatr. 167, 751–755. 10.1007/s00431-007-0582-0 17704945

[B19] Chen Y.-t. Tai C.-Y. (2017). μ2-Dependent Endocytosis of N-Cadherin Is Regulated by β-catenin to Facilitate Neurite Outgrowth. Traffic 18, 287–303. 10.1111/tra.12473 28224728

[B20] Cheng C. Gokhin D. S. Nowak R. B. Fowler V. M. (2016a). Sequential Application of Glass Coverslips to Assess the Compressive Stiffness of the Mouse Lens: Strain and Morphometric Analyses. J. Vis. Exp. 2016 (111), 53986. 10.3791/53986 PMC4942030 27166880

[B21] Cheng C. Nowak R. B. Biswas S. K. Lo W.-K. FitzGerald P. G. Fowler V. M. (2016b). Tropomodulin 1 Regulation of Actin Is Required for the Formation of Large Paddle Protrusions between Mature Lens Fiber Cells. Investig. Ophthalmol. Vis. Sci. 57, 4084–4099. 10.1167/iovs.16-19949 27537257 PMC4986768

[B22] Cvekl A. Ashery-Padan R. (2014). The Cellular and Molecular Mechanisms of Vertebrate Lens Development. Development 141, 4432–4447. 10.1242/dev.107953 25406393 PMC4302924

[B23] Elia L. P. Yamamoto M. Zang K. Reichardt L. F. (2006). p120 Catenin Regulates Dendritic Spine and Synapse Development through Rho-Family GTPases and Cadherins. Neuron 51, 43–56. 10.1016/j.neuron.2006.05.018 16815331 PMC2587166

[B24] Fang X. Ji H. Kim S.-W. Park J.-I. Vaught T. G. Anastasiadis P. Z. (2004). Vertebrate Development Requires ARVCF and P120 Catenins and Their Interplay with RhoA and Rac. J. Cell. Biol. 165, 87–98. 10.1083/jcb.200307109 15067024 PMC2172091

[B25] Fisher R. F. (1973). Human Lens Fibre Transparency and Mechanical Stress. Exp. Eye Res. 16, 41–49. 10.1016/0014-4835(73)90235-2 4718700

[B26] Frederikse P. H. Kasinathan C. Kleiman N. J. (2012). Parallels between Neuron and Lens Fiber Cell Structure and Molecular Regulatory Networks. Dev. Biol. 368, 255–260. 10.1016/j.ydbio.2012.05.022 22641011

[B27] Fudge D. S. McCuaig J. V. Van Stralen S. Hess J. F. Wang H. Mathias R. T. (2011). Intermediate Filaments Regulate Tissue Size and Stiffness in the Murine Lens. Investig. Ophthalmol. Vis. Sci. 52, 3860–3867. 10.1167/iovs.10-6231 21345981 PMC3109061

[B28] Gao J. Sun X. Moore L. C. White T. W. Brink P. R. Mathias R. T. (2011). Lens Intracellular Hydrostatic Pressure Is Generated by the Circulation of Sodium and Modulated by Gap Junction Coupling. J. Gen. Physiol. 137, 507–520. 10.1085/jgp.201010538 21624945 PMC3105514

[B29] Gokhin D. S. Nowak R. B. Kim N. E. Arnett E. E. Chen A. C. Sah R. L. (2012). Tmod1 and CP49 Synergize to Control the Fiber Cell Geometry, Transparency, and Mechanical Stiffness of the Mouse Lens. Plos One 7, e48734. 10.1371/journal.pone.0048734 23144950 PMC3492431

[B30] Gokturk B. Topcu-Yilmaz P. Bozkurt B. Yildirim M. S. Guner S. N. Sayar E. H. (2016). Ocular Findings in Children with 22q11.2 Deletion Syndrome. J. Pediatr. Ophthalmol. Strabismus 53, 218–222. 10.3928/01913913-20160427-01 27182748

[B31] Gonen T. Cheng Y. Kistler J. Walz T. (2004). Aquaporin-0 Membrane Junctions Form upon Proteolytic Cleavage. J. Mol. Biol. 342, 1337–1345. 10.1016/j.jmb.2004.07.076 15351655

[B32] Gu S. Biswas S. Rodriguez L. Li Z. Li Y. Riquelme M. A. (2019). Connexin 50 and AQP0 Are Essential in Maintaining Organization and Integrity of Lens Fibers. Investig. Ophthalmol. Vis. Sci. 60, 4021–4032. 10.1167/iovs.18-26270 31560767 PMC6779290

[B33] Houssin N. S. Martin J. B. Coppola V. Yoon S. O. Plageman T. F. (2020). Formation and Contraction of Multicellular Actomyosin Cables Facilitate Lens Placode Invagination. Dev. Biol. 462, 36–49. 10.1016/j.ydbio.2020.02.014 32113830 PMC7225080

[B34] Hu Z. Shi W. Riquelme M. A. Shi Q. Biswas S. Lo W. K. (2017). Connexin 50 Functions as an Adhesive Molecule and Promotes Lens Cell Differentiation. Sci. Rep. 7, 5298. 10.1038/s41598-017-05647-9 28706245 PMC5509658

[B35] Huff J. (2015). The Airyscan Detector from ZEISS: Confocal Imaging with Improved Signal-To-Noise Ratio and Super-resolution. Nat. Methods 12 , i–ii. 10.1038/nmeth.f.388

[B36] Ishiyama N. Lee S.-H. Liu S. Li G.-Y. Smith M. J. Reichardt L. F. (2010). Dynamic and Static Interactions between P120 Catenin and E-Cadherin Regulate the Stability of Cell-Cell Adhesion. Cell. 141, 117–128. 10.1016/j.cell.2010.01.017 20371349

[B37] Jun G. Moncaster J. A. Koutras C. Seshadri S. Buros J. McKee A. C. (2012). δ-Catenin Is Genetically and Biologically Associated with Cortical Cataract and Future Alzheimer-Related Structural and Functional Brain Changes. Plos One 7, e43728. 10.1371/journal.pone.0043728 22984439 PMC3439481

[B38] Khairallah M. Kahloun R. Bourne R. Limburg H. Flaxman S. R. Jonas J. B. (2015). Number of People Blind or Visually Impaired by Cataract Worldwide and in World Regions, 1990 to 2010. Investig. Ophthalmol. Vis. Sci. 56, 6762–6769. 10.1167/iovs.15-17201 26567788

[B39] Kumari S. S. Gandhi J. Mustehsan M. H. Eren S. Varadaraj K. (2013). Functional Characterization of an AQP0 Missense Mutation, R33C, that Causes Dominant Congenital Lens Cataract, Reveals Impaired Cell-To-Cell Adhesion. Exp. Eye Res. 116, 371–385. 10.1016/j.exer.2013.09.019 24120416 PMC3864651

[B40] Lachke S. A. Higgins A. W. Inagaki M. Saadi I. Xi Q. Long M. (2012). The Cell Adhesion Gene PVRL3 Is Associated with Congenital Ocular Defects. Hum. Genet. 131, 235–250. 10.1007/s00439-011-1064-z 21769484 PMC3279124

[B41] Lang R. A. Herman K. Reynolds A. B. Hildebrand J. D. Plageman T. F. (2014). p120-catenin-dependent Junctional Recruitment of Shroom3 Is Required for Apical Constriction during Lens Pit Morphogenesis. Development 141, 3177–3187. 10.1242/dev.107433 25038041 PMC4197547

[B42] Li Y.-J. Goh L. Khor C.-C. Fan Q. Yu M. Han S. (2011). Genome-Wide Association Studies Reveal Genetic Variants in CTNND2 for High Myopia in Singapore Chinese. Ophthalmology 118, 368–375. 10.1016/j.ophtha.2010.06.016 21095009 PMC3052933

[B43] Lo W.-K. Biswas S. K. Brako L. Shiels A. Gu S. Jiang J. X. (2014). Aquaporin-0 Targets Interlocking Domains to Control the Integrity and Transparency of the Eye Lens. Investig. Ophthalmol. Vis. Sci. 55, 1202–1212. 10.1167/iovs.13-13379 24458158 PMC3941616

[B44] Lo W.-K. Shaw A. P. Wen X.-J. (1997). Actin Filament Bundles in Cortical Fiber Cells of the Rat Lens. Exp. eye Res. 65, 691–701. 10.1006/exer.1997.0375 9367649

[B45] Lo W. K. (1988). Adherens Junctions in the Ocular Lens of Various Species: Ultrastructural Analysis with an Improved Fixation. Cell. Tissue Res. 254, 31–40. 10.1007/BF00220014 3143480

[B46] Logan C. M. Bowen C. J. Menko A. S. (2017a). Induction of Immune Surveillance of the Dysmorphogenic Lens. Sci. Rep. 7, 16235. 10.1038/s41598-017-16456-5 29176738 PMC5701161

[B47] Logan C. M. Rajakaruna S. Bowen C. Radice G. L. Robinson M. L. Menko A. S. (2017b). N-cadherin Regulates Signaling Mechanisms Required for Lens Fiber Cell Elongation and Lens Morphogenesis. Dev. Biol. 428, 118–134. 10.1016/j.ydbio.2017.05.022 28552735 PMC5524459

[B48] Maddala R. Rao P. V. (2017). Switching of α-Catenin from Epithelial to Neuronal Type during Lens Epithelial Cell Differentiation. Investig. Ophthalmol. Vis. Sci. 58, 3445–3455. 10.1167/iovs.17-21539 28692740 PMC5505122

[B7] Maisel H. Atreya P. L. (1990). N-Cadherin Detected in the Membrane Fraction of Lens Fiber Cells. Experientia 46, 222–223. 10.1007/BF02027322 2303131

[B49] Mansour A. M. Goldberg R. B. Wang F. M. Shprintzen R. J. (1987). Ocular Findings in the Velo-Cardio-Facial Syndrome. J. Pediatr. Ophthalmol. Strabismus 24, 263–266. 10.3928/0191-3913-19870901-16 3681616

[B50] McCrea P. D. Park J.-i. (2007). Developmental Functions of the P120-Catenin Sub-family. Biochimica Biophysica Acta (BBA) - Mol. Cell. Res. 1773, 17–33. 10.1016/j.bbamcr.2006.06.009 16942809

[B51] Menke A. Giehl K. (2012). Regulation of Adherens Junctions by Rho GTPases and P120-Catenin. Archives Biochem. Biophysics 524, 48–55. 10.1016/j.abb.2012.04.019 22583808

[B52] Michael R. Barraquer R. I. Willekens B. van Marle J. Vrensen G. F. J. M. (2008). Morphology of Age-Related Cuneiform Cortical Cataracts: The Case for Mechanical Stress. Vis. Res. 48, 626–634. 10.1016/j.visres.2007.12.005 18221767

[B53] Miyashita Y. Ozawa M. (2007). Increased Internalization of P120-Uncoupled E-Cadherin and a Requirement for a Dileucine Motif in the Cytoplasmic Domain for Endocytosis of the Protein. J. Biol. Chem. 282, 11540–11548. 10.1074/jbc.m608351200 17298950

[B54] Niessen C. M. Gottardi C. J. (2008). Molecular Components of the Adherens Junction. Biochimica Biophysica Acta (BBA) - Biomembr. 1778, 562–571. 10.1016/j.bbamem.2007.12.015 PMC2276178 18206110

[B55] Pau H. (2006). Cortical and Subcapsular Cataracts: Significance of Physical Forces. Ophthalmologica 220, 1–5. 10.1159/000089267 16374041

[B56] Pieters T. van Hengel J. van Roy F. (2012). Functions of P120ctn in Development and Disease. Front. Biosci. 17, 760–783. 10.2741/3956 22201773

[B57] Pontoriero G. F. Smith A. N. Miller L.-A. D. Radice G. L. West-Mays J. A. Lang R. A. (2009). Co-operative Roles for E-Cadherin and N-Cadherin during Lens Vesicle Separation and Lens Epithelial Cell Survival. Dev. Biol. 326, 403–417. 10.1016/j.ydbio.2008.10.011 18996109 PMC3408230

[B58] Rivero-Gutiérrez B. Anzola A. Martínez-Augustin O. de Medina F. S. (2014). Stain-free Detection as Loading Control Alternative to Ponceau and Housekeeping Protein Immunodetection in Western Blotting. Anal. Biochem. 467, 1–3. 10.1016/j.ab.2014.08.027 25193447

[B59] Shiels A. Bassnett S. (1996). Mutations in the Founder of the MIP Gene Family Underlie Cataract Development in the Mouse. Nat. Genet. 12, 212–215. 10.1038/ng0296-212 8563764

[B60] Shiels A. Bassnett S. Varadaraj K. Mathias R. Al-Ghoul K. Kuszak J. (2001). Optical Dysfunction of the Crystalline Lens in Aquaporin-0-Deficient Mice. Physiol. Genomics 7, 179–186. 10.1152/physiolgenomics.00078.2001 11773604

[B61] Shprintzen R. J. (2008). Velo-cardio-facial Syndrome: 30 Years of Study. Dev. Disabil. Res. Revs 14, 3–10. 10.1002/ddrr.2 18636631 PMC2805186

[B62] Sindhu Kumari S. Gupta N. Shiels A. Fitzgerald P. G. Menon A. G. Mathias R. T. (2015). Role of Aquaporin 0 in Lens Biomechanics. Biochem. Biophysical Res. Commun. 462, 339–345. 10.1016/j.bbrc.2015.04.138 PMC4461499 25960294

[B63] Smith A. N. Miller L.-A. D. Song N. Taketo M. M. Lang R. A. (2005). The Duality of β-catenin Function: A Requirement in Lens Morphogenesis and Signaling Suppression of Lens Fate in Periocular Ectoderm. Dev. Biol. 285, 477–489. 10.1016/j.ydbio.2005.07.019 16102745

[B64] Straub B. K. Boda J. Kuhn C. Schnoelzer M. Korf U. Kempf T. (2003). A Novel Cell-Cell Junction System: the Cortex Adhaerens Mosaic of Lens Fiber Cells. J. Cell. Sci. 116, 4985–4995. 10.1242/jcs.00815 14625392

[B65] Sun W. Xu J. Gu Y. Du C. (2021). The Relationship between Major Intrinsic Protein Genes and Cataract. Int. Ophthalmol. 41, 375–387. 10.1007/s10792-020-01583-2 32920712

[B66] Tai C.-Y. Mysore S. P. Chiu C. Schuman E. M. (2007). Activity-regulated N-Cadherin Endocytosis. Neuron 54, 771–785. 10.1016/j.neuron.2007.05.013 17553425

[B67] Togashi H. Abe K. Mizoguchi A. Takaoka K. Chisaka O. Takeichi M. (2002). Cadherin Regulates Dendritic Spine Morphogenesis. Neuron 35, 77–89. 10.1016/s0896-6273(02)00748-1 12123610

[B68] Truong Quang B.-A. Mani M. Markova O. Lecuit T. Lenne P.-F. (2013). Principles of E-Cadherin Supramolecular Organization *In Vivo* . Curr. Biol. 23, 2197–2207. 10.1016/j.cub.2013.09.015 24184100

[B69] Truscott R. J. W. Friedrich M. G. (2019). Molecular Processes Implicated in Human Age-Related Nuclear Cataract. Investig. Ophthalmol. Vis. Sci. 60, 5007–5021. 10.1167/iovs.19-27535 31791064 PMC7043214

[B70] Varadaraj K. Kumari S. (2019). Deletion of Seventeen Amino Acids at the C-Terminal End of Aquaporin 0 Causes Distortion Aberration and Cataract in the Lenses of AQP0ΔC/ΔCMice. Investig. Ophthalmol. Vis. Sci. 60, 858–867. 10.1167/iovs.18-26378 30821811 PMC6397018

[B71] Varadaraj K. Kumari S. S. (2018). Molecular Mechanism of Aquaporin 0-induced Fiber Cell to Fiber Cell Adhesion in the Eye Lens. Biochem. biophysical Res. Commun. 506, 284–289. 10.1016/j.bbrc.2018.10.066 PMC6223623 30348525

[B72] Vrensen G. Willekens B. (1990). Biomicroscopy and Scanning Electron Microscopy of Early Opacities in the Aging Human Lens. Investig. Ophthalmol. Vis. Sci. 31, 1582–1591. 2387688

[B73] Wang E. Geng A. Maniar A. M. Mui B. W. H. Gong X. (2016). Connexin 50 Regulates Surface Ball-and-Socket Structures and Fiber Cell Organization. Investig. Ophthalmol. Vis. Sci. 57, 3039–3046. 10.1167/iovs.16-19521 27281269 PMC4913802

[B74] White T. W. Goodenough D. A. Paul D. L. (1998). Targeted Ablation of Connexin50 in Mice Results in Microphthalmia and Zonular Pulverulent Cataracts. J. Cell. Biol. 143, 815–825. 10.1083/jcb.143.3.815 9813099 PMC2148149

[B75] Wu Y. Kanchanawong P. Zaidel-Bar R. (2015). Actin-Delimited Adhesion-independent Clustering of E-Cadherin Forms the Nanoscale Building Blocks of Adherens Junctions. Dev. Cell. 32, 139–154. 10.1016/j.devcel.2014.12.003 25600236

[B76] Xiao K. Garner J. Buckley K. M. Vincent P. A. Chiasson C. M. Dejana E. (2005). p120-Catenin Regulates Clathrin-dependent Endocytosis of VE-Cadherin. MBoC 16, 5141–5151. 10.1091/mbc.e05-05-0440 16120645 PMC1266414

[B77] Xie Z. Photowala H. Cahill M. E. Srivastava D. P. Woolfrey K. M. Shum C. Y. (2008). Coordination of Synaptic Adhesion with Dendritic Spine Remodeling by AF-6 and Kalirin-7. J. Neurosci. 28, 6079–6091. 10.1523/jneurosci.1170-08.2008 18550750 PMC2727754

[B78] Yap A. S. Gomez G. A. Parton R. G. (2015). Adherens Junctions Revisualized: Organizing Cadherins as Nanoassemblies. Dev. Cell. 35, 12–20. 10.1016/j.devcel.2015.09.012 26460944

[B79] Yap A. S. Niessen C. M. Gumbiner B. M. (1998). The Juxtamembrane Region of the Cadherin Cytoplasmic Tail Supports Lateral Clustering, Adhesive Strengthening, and Interaction with P120ctn. J. Cell. Biol. 141, 779–789. 10.1083/jcb.141.3.779 9566976 PMC2132752

