# Supplementary material for: Arvcf Dependent Adherens Junction Stability is Required to Prevent Age-Related Cortical Cataracts
Source: Front Cell Dev Biol. 2022 Jul 6;10:840129. doi: 10.3389/fcell.2022.840129 (PMC9297370; doi:10.3389/fcell.2022.840129)
Supplement: Supplementary file 1 [file Presentation1.pdf]

**Supplemental Figure 1:** A) Diagram of the elements inserted into the Arvcf locus as a result of gene targeting. The gray boxes indicate the exons and the blue half arrows indicate the approximate location of genotyping primers used to identify the targeted allele. B) Representative results from genotyping following PCR amplification. C) The table represents the number of embryos with the indicated genotypes collected from timed matings of heterozygous crossings. D-I) Imunofluorescent labeling of F-actin and nuclei (D-F) or Arvcf and nuclei (G-I) of transverse cryosections lenses from embryos with the indicated genotype. Note that no obvious ocular phenotype is observed in Arvcf null embryos despite the absence of detectable protein. Scale bars=50microns.

**Supplemental Figure 2:** A-B) Images of lenses dissected from control (A) or Arvcf null lenses (B-C). The arrowheads demonstrate regions of cortical spoking. The white lines in B represent a transparent region that is superficial to the opaque region. C) A higher magnification of an Arvcf-/- lens with a disruption at the anterior pole that occurs along a suture line. D-E) Examples of lenses at the indicated ages dissected from control or Arvcf-/- animals that have been placed on an electron microscopy grid (D) or an ink-printer generated grid (E). F) Box and whisker plots that describe the quantitative data depicted in Figure 2. The line within each box represents the median value, the boxes span the first and third quartiles, the x symbol represents the mean, the whiskers represent the range of all values not considered outliers, and the circles represent the outlier values that are outside 1.5 times the interquartile range.

**Supplemental Figure 3:** A) A histological equatorial cryosection of a control and Arvcf-deficient lens was immunolabeled for αB-crystallin and WGA. The image is focused on a region near the lens nucleus where significant disruptions in fiber cell occur. The asterisks mark unusually large cells that are bounded by membrane (red) but filled with crystallin-containing cytoplasm (green). Note that this indicates that these regions are not intercellular breaks but rather abnormally large regions of cytoplasm. B) The entire polyacrylamide gel following SDS-PAGE of lysates from control and Arvcf mutant lens lysates labeled for total protein lysate found in figure 3H is on the left. On the right is the entire western blot of the polyacrylamide gel depicted to the left that has been immunolabeled for Arvcf protein. C-D) Examples of additional western blots demonstrating the reduction of Arvcf protein in *Arvcf* $^{-/-}$ lens lysates. The white hatched boxes in D are magnified in panel C. E) Equatorial histological sections of control and Arvcf mutant lens fiber cells found within 50microns of the surface co-immunolabeled with p120-catenin (green) and β-catenin (red). Note that p120-catenin is not strongly localized to lens fiber cell membranes in control lenses but is more apparent in the lens fiber cell membranes of mutant lenses. F-J) Box and whisker plots that describe the quantitative data depicted in Figures 4-5. The line within each box represents the median value, the boxes span the first and third quartiles, the x symbol represents the mean, the whiskers represent the range of all values not considered outliers, and the circles represent the outlier values that are outside 1.5 times the interquartile range. Scale bars=10microns

**Supplemental Figure 4:** A-B) Lower and higher magnification SEM images of a whole bisected lens or a small region of the lens approximately 100 microns from the surface from control (A) or Arvcf deficient lenses (B). In panels A' and B' a single lens fiber cell was pseudocolored green to visualize their elongated nature. The scale bars in A and B = 300 microns and the scale bars in A' and B' = 5 microns. C) Image of an individual interlocking protrusion from an *Arvcf* $^{-/-}$ lens immunofluorescently co-labeled with antibodies specific for β-catenin (green C") or aquaporin-0 (blue C'). The diagram to the right indicates the strategy for comparing immunofluorescent images of individual protrusions. The numbers represent individual pixels labeled by position. The pixels at the same location (those that have the same position number) were compared among each protrusion image by quantifying the mean. These means were used to generate the average image in Fig. 7C,D,I,J and used to perform the subsequent statistical analyses. D) A single wild-type lens fiber cell immunofluorescently labeled with an N-cadherin and

aquaporin-0 antibody. Note that this lens fiber cell was prepared by pulling individual fiber cells apart. E) Magnified images of the regions indicated by boxes in panel D. Yellow asterisks mark cellular protrusion outlines that lie on the surface of lens fiber cells. These outlines are well delineated by both N-cadherin and Aqp0 localization and are thought to correspond to the “pocket” of an interlocking protrusion from a neighboring cell. Note that these are typically outlined well and are unaffected by dissection. This stands in contrast to the protrusions that extend into space following manual dissection (arrowheads). These are readily visualized by Aqp0 localization but not N-cadherin. F) A group of wild-type lens fiber cells immunofluorescently labeled for Arvcf and N-cadherin were imaged without dissecting them from each other but have a slight separation. The yellow boxed regions are magnified in the small panels to the right. Note that N-cadherin appears to have a similar localization pattern to their neighboring cells along the margins of paddle regions. This also apparent in interlocking protrusions (bottom right panel, asterisks). Scalebars in panels D-F = 2000 nm
