# Supplementary figures and images for: Arvcf Dependent Adherens Junction Stability is Required to Prevent Age-Related Cortical Cataracts
Source: Front Cell Dev Biol. 2022 Jul 6;10:840129. doi: 10.3389/fcell.2022.840129 (PMC9297370; doi:10.3389/fcell.2022.840129)

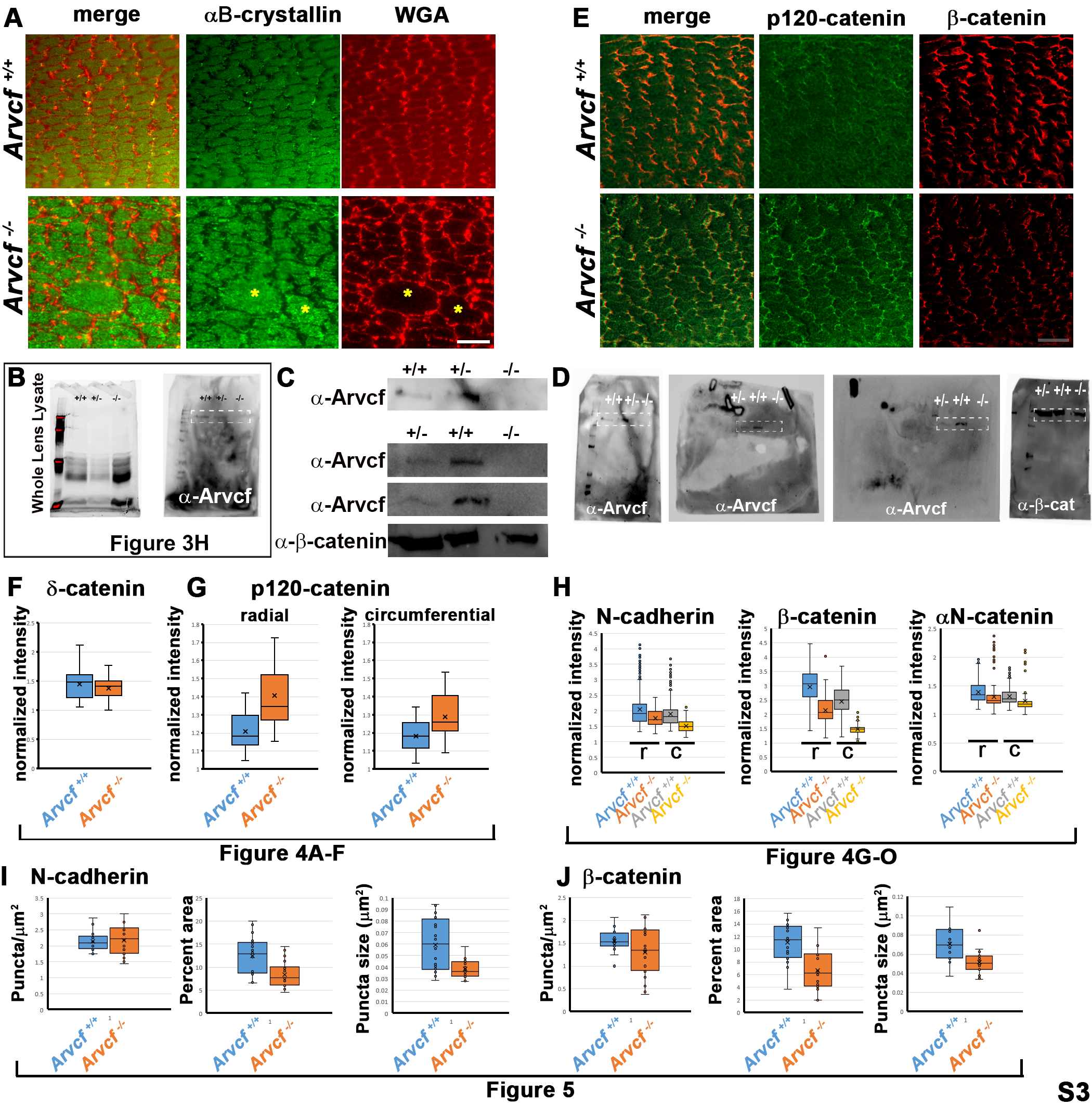

Supplement: Supplementary file 2 [file Image3.tif]

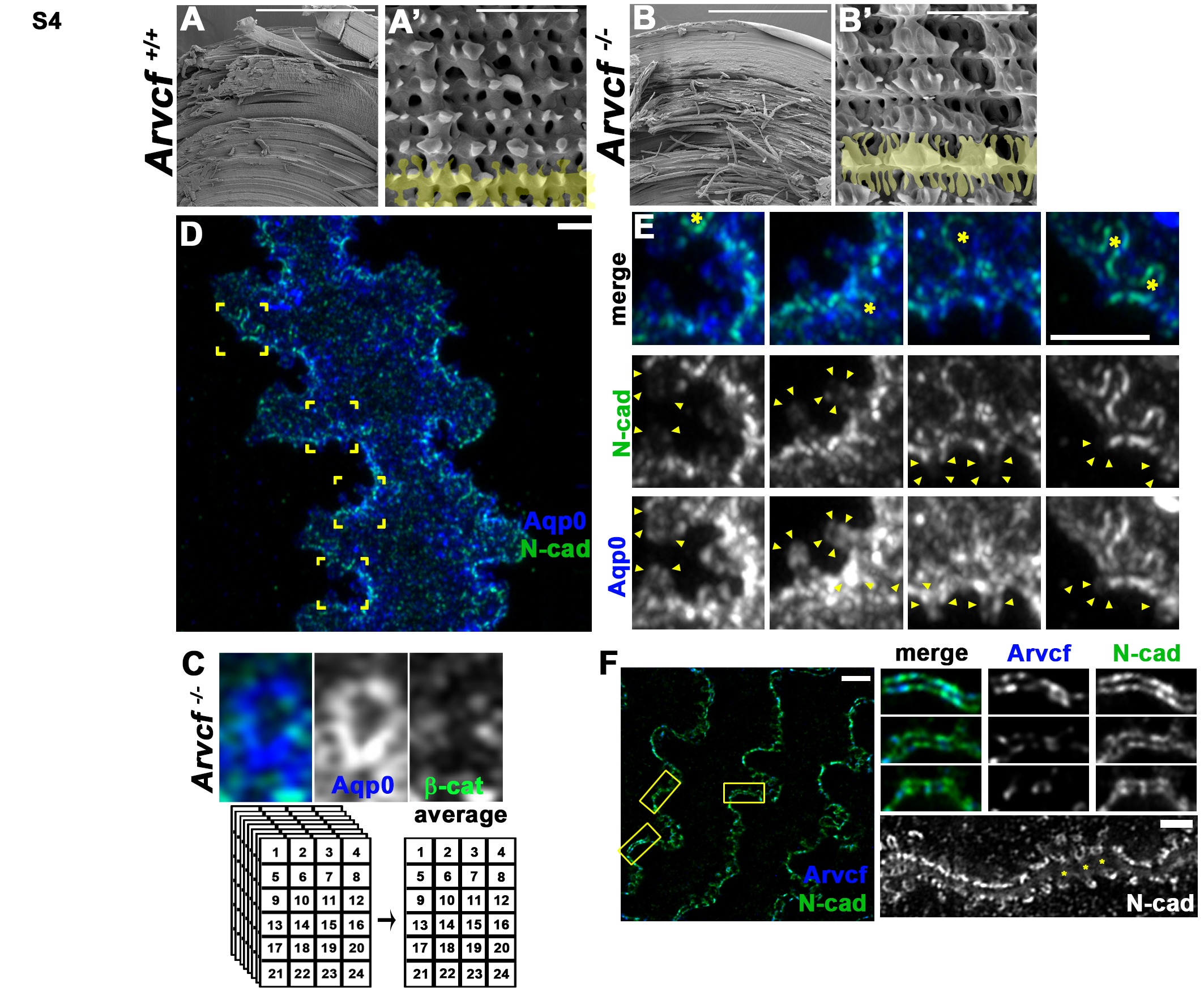

Supplement: Supplementary file 3 [file Image4.tif]

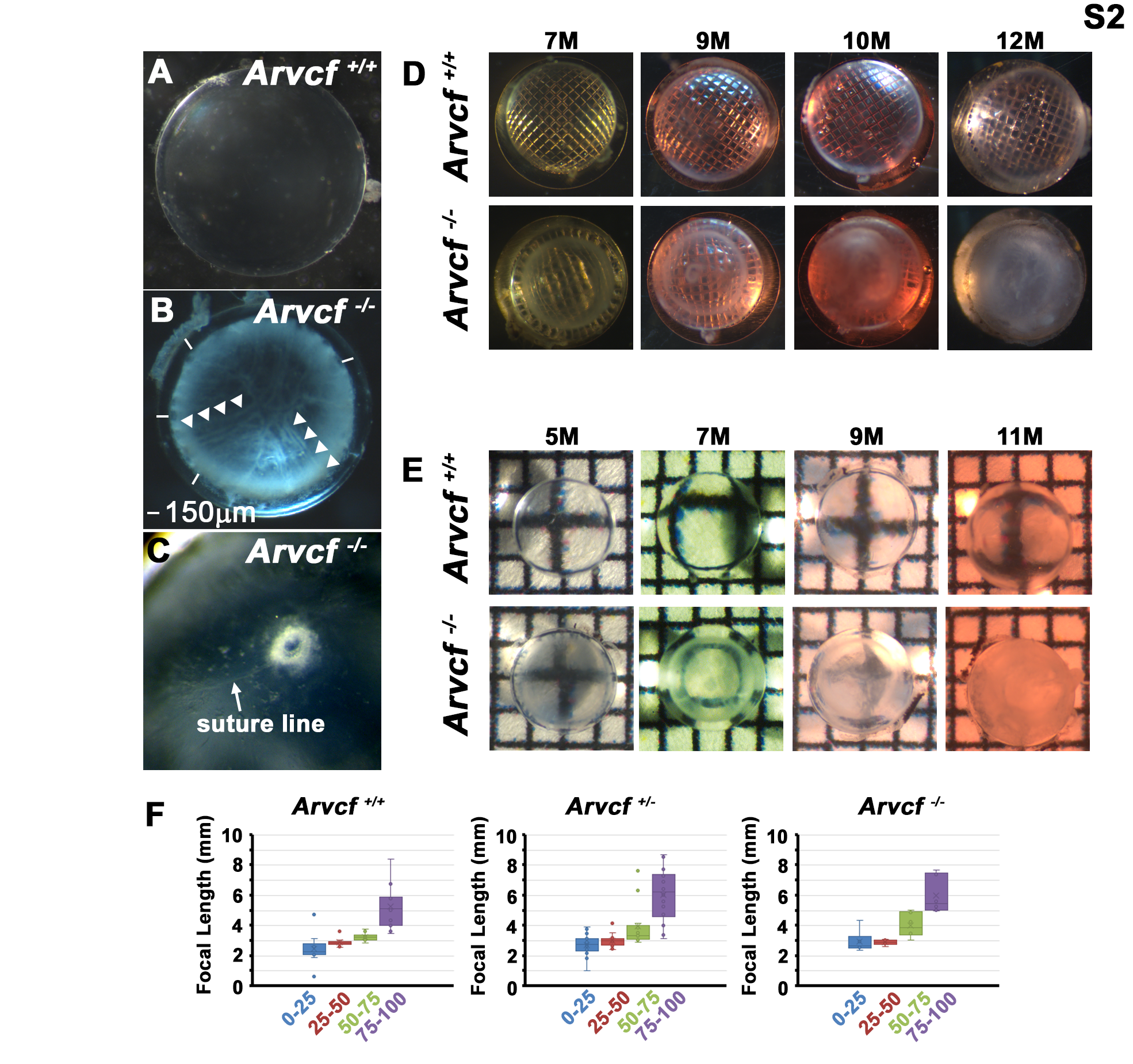

Supplement: Supplementary file 4 [file Image2.tif]

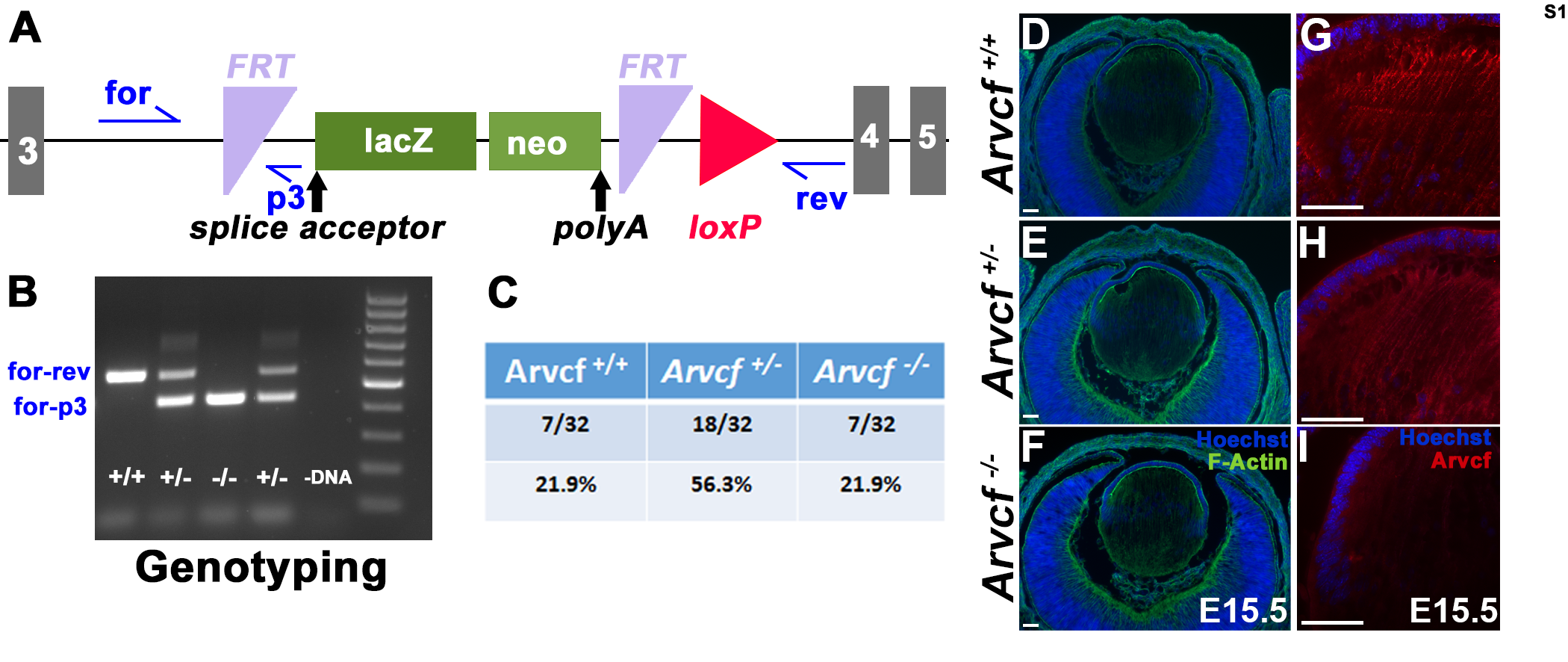

Supplement: Supplementary file 5 [file Image1.tif]
